# Methylotrophy in the Mire: direct and indirect routes for methane production in thawing permafrost

**DOI:** 10.1128/msystems.00698-23

**Published:** 2023-12-08

**Authors:** Jared B. Ellenbogen, Mikayla A. Borton, Bridget B. McGivern, Dylan R. Cronin, David W. Hoyt, Viviana Freire-Zapata, Carmody K. McCalley, Ruth K. Varner, Patrick M. Crill, Richard A. Wehr, Jeffrey P. Chanton, Ben J. Woodcroft, Malak M. Tfaily, Gene W. Tyson, Virginia I. Rich, Kelly C. Wrighton

**Affiliations:** 1 Department of Soil and Crop Science, Colorado State University, Fort Collins, Colorado, USA; 2 Department of Microbiology, The Ohio State University, Columbus, Ohio, USA; 3 Environmental Molecular Sciences Laboratory, Earth and Biological Sciences Division, Pacific Northwest National Laboratory, Richland, Washington, USA; 4 Department of Environmental Science, University of Arizona, Tucson, Arizona, USA; 5 Gosnell School of Life Sciences, Rochester Institute of Technology, Rochester, New York, USA; 6 Department of Earth Sciences and Institute for the Study of Earth, Oceans and Space, University of New Hampshire, Durham, New Hampshire, USA; 7 Department of Geological Sciences, Bolin Center for Climate Research, Stockholm University, Stockholm, Sweden; 8 Department of Ecology and Evolutionary Biology, University of Arizona, Tucson, Arizona, USA; 9 Earth Ocean and Atmospheric Sciences, Florida State University, Tallahassee, Florida, USA; 10 Centre for Microbiome Research, School of Biomedical Sciences, Queensland University of Technology (QUT), Translational Research Institute, Woolloongabba, Queensland, Australia; University of East Anglia, Norwich, United Kingdom

**Keywords:** methanogenesis, methylotrophy, Stordalen Mire, EMERGE Biology Integration Institute

## Abstract

While wetlands are major sources of biogenic methane (CH_4_), our understanding of resident microbial metabolism is incomplete, which compromises the prediction of CH_4_ emissions under ongoing climate change. Here, we employed genome-resolved multi-omics to expand our understanding of methanogenesis in the thawing permafrost peatland of Stordalen Mire in Arctic Sweden. In quadrupling the genomic representation of the site’s methanogens and examining their encoded metabolism, we revealed that nearly 20% of the metagenome-assembled genomes (MAGs) encoded the potential for methylotrophic methanogenesis. Further, 27% of the transcriptionally active methanogens expressed methylotrophic genes; for *Methanosarcinales* and *Methanobacteriales* MAGs, these data indicated the use of methylated oxygen compounds (e.g., methanol), while for *Methanomassiliicoccales*, they primarily implicated methyl sulfides and methylamines. In addition to methanogenic methylotrophy, >1,700 bacterial MAGs across 19 phyla encoded anaerobic methylotrophic potential, with expression across 12 phyla. Metabolomic analyses revealed the presence of diverse methylated compounds in the Mire, including some known methylotrophic substrates. Active methylotrophy was observed across all stages of a permafrost thaw gradient in Stordalen, with the most frozen non-methanogenic palsa found to host bacterial methylotrophy and the partially thawed bog and fully thawed fen seen to house both methanogenic and bacterial methylotrophic activities. Methanogenesis across increasing permafrost thaw is thus revised from the sole dominance of hydrogenotrophic production and the appearance of acetoclastic at full thaw to consider the co-occurrence of methylotrophy throughout. Collectively, these findings indicate that methanogenic and bacterial methylotrophy may be an important and previously underappreciated component of carbon cycling and emissions in these rapidly changing wetland habitats.

**IMPORTANCE:**

Wetlands are the biggest natural source of atmospheric methane (CH_4_) emissions, yet we have an incomplete understanding of the suite of microbial metabolism that results in CH_4_ formation. Specifically, methanogenesis from methylated compounds is excluded from all ecosystem models used to predict wetland contributions to the global CH_4_ budget. Though recent studies have shown methylotrophic methanogenesis to be active across wetlands, the broad climatic importance of the metabolism remains critically understudied. Further, some methylotrophic bacteria are known to produce methanogenic by-products like acetate, increasing the complexity of the microbial methylotrophic metabolic network. Prior studies of Stordalen Mire have suggested that methylotrophic methanogenesis is irrelevant *in situ* and have not emphasized the bacterial capacity for metabolism, both of which we countered in this study. The importance of our findings lies in the significant advancement toward unraveling the broader impact of methylotrophs in wetland methanogenesis and, consequently, their contribution to the terrestrial global carbon cycle.

## INTRODUCTION

Wetlands are the largest natural source of atmospheric methane (CH_4_) emissions (1, 2), with those found in permafrost zones of specific concern to the global CH_4_ budget due to their sensitivity to a warming climate. Climate change-induced permafrost thaw is anticipated to make large quantities of previously frozen near-surface carbon available to soil microbiota over the next century, which could accelerate CH_4_ production and release (3). Methanogenic archaea are the primary biological producers of CH_4_ in soils via three distinct pathways of methanogenesis (4). In saturated soils, there is wide appreciation for hydrogenotrophic and acetoclastic pathways from microorganisms that utilize H_2_/carbon dioxide (CO_2_) and acetate, respectively, to form CH_4_ (4, 5). The methylotrophic pathway, which allows organisms to use and reduce methylated compounds for CH_4_ production (6, 7), is far less studied though appreciation of it is growing. This knowledge gap contributes to the fact that contemporary process-based biogeochemical models account only for hydrogenotrophic and acetoclastic microbial CH_4_ production in climate-relevant soil systems (8).

In contrast to historical paradigms, recent genomic insights have greatly expanded our understanding of the distribution and activity of methylotrophic methanogens, especially in saturated, high-CH_4_-emitting soils (9–17). Similarly, biochemical and physiological efforts have expanded the suite of substrates known to be utilized by methylotrophic methanogens (18–24). These methanogens catabolize methylated compounds via oxygen-sensitive substrate-specific so-called three-component methyltransferase systems (also frequently referred to as corrinoid-dependent methyltransferase systems) (6, 7). This gene content information can be used to physiologically classify methylotrophic methanogens as using one or more of three major substrate categories: methylated amines like trimethylamine or glycine betaine (methyl-N) (20, 25–27), methylated sulfides like dimethyl sulfide (methyl-S) (23, 28), and methylated oxygen compounds like methanol (methyl-O) (18, 19, 29). Moreover, methylotrophic methanogens can be obligate, meaning they only utilize methylated compounds to produce CH_4_, or facultative, meaning they encode and can express multiple pathways for CH_4_ production (10, 24). In addition to methanogens, some anaerobic bacteria can use similar corrinoid-dependent methyltransferase systems in a non-methanogenic mechanism for both carbon assimilation and energy generation (30–38). In summary, while diverse efforts have increased knowledge of anaerobic methylotrophic metabolism, these types of metabolism remain undercharacterized in climate-relevant soil systems.

In this study, we used a genome-resolved, multi-omic approach to profile the potential for anaerobic, corrinoid-dependent methylotrophic metabolism across a rapidly thawing CH_4_-emitting permafrost peatland (39, 40) located in Arctic Sweden. This offered us a unique opportunity to study this metabolism across a discontinuous permafrost thaw gradient encompassing three distinct habitat types—palsa, bog, and fen—at multiple depths within each (Fig. S1). Prior characterization of the soil microbiota across this thaw gradient has included analysis of the native methanogen community, focusing primarily on the dominant hydrogenotrophic *Methanoflorens stordalenmirensis* [genus “Bog-38” in the Genome Taxonomy Database (GTDB)] (41–43). Here, with metagenomic sequencing of more samples, we expand the catalog of methanogens for the site, offering new opportunities for resolved physiological characterization of methanogenic pathways. Using a combination of metagenome, metabolite, and metatranscriptome data, we demonstrate anaerobic methylotrophy to be an underappreciated part of the CH_4_ cycle in Stordalen Mire and suggest the *in situ* methylotrophic metabolic network to be of previously unrecognized complexity. These findings let us build a new conceptual model of how methylotrophic metabolism can directly and indirectly modulate CH_4_ fluxes across the Mire.

## RESULTS

### One-fifth of Stordalen Mire’s diverse methanogens, spanning three orders, encode methylotrophic methanogenesis

Field sampling from 2010 to 2017 has yielded an extensive microbial metagenome-assembled genome (MAG) database from Stordalen Mire (43). Of the MAGs with GTDB-assigned taxonomy of archaea, functional analyses confirmed that 367 (Table S1 tab Methanogen_Genome_Info) encoded the potential for methanogenesis via screening with the microbial genome annotation software DRAM (Distilled and Refined Annotation of Metabolism) (44) for the presence of a variety of methanogenesis genes, including those encoding the Mcr and Hdr complexes (Fig. S2A; Table S2 tab Gene_KO_information). This roughly quadrupled the known methanogen MAGs so far known from this site, with 23% of the methanogens having been previously reported in Woodcroft et al. (43).

We next curated the metabolic potential of these MAGs, revealing a diversity of substrate-specific methanogenic physiology measured here within the community (Fig. S2; Table S2 tab MAG_Physiology_Summary). Methanogenic MAGs were screened with DRAM for indicators of hydrogenotrophic, acetoclastic, and methylotrophic methanogenesis (5, 6, 45–48) (Fig. S2B and C). The *Methanoflorens* and *Methanomicrobiales* were designated hydrogenotrophic, the *Methanotrichales* acetoclastic, and the *Methanomassiliicoccales* methylotrophic. Notably, many *Methanosarcinales* were found to encode all modes of methanogenesis, and the *Methanobacteriales* were found to encode both hydrogenotrophic and methylotrophic potential. While this DRAM-enabled analysis suggested greater methylotrophic potential among the Stordalen methanogenic community than previously known, much of the known physiological and biochemical diversity of methylotrophy is absent from databases used by genome annotation software (e.g., KEGG). Thus, we opted to manually curate the methylotrophic potential of these MAGs to better assess their substrate-specific metabolic potential.

**Fig 1:**
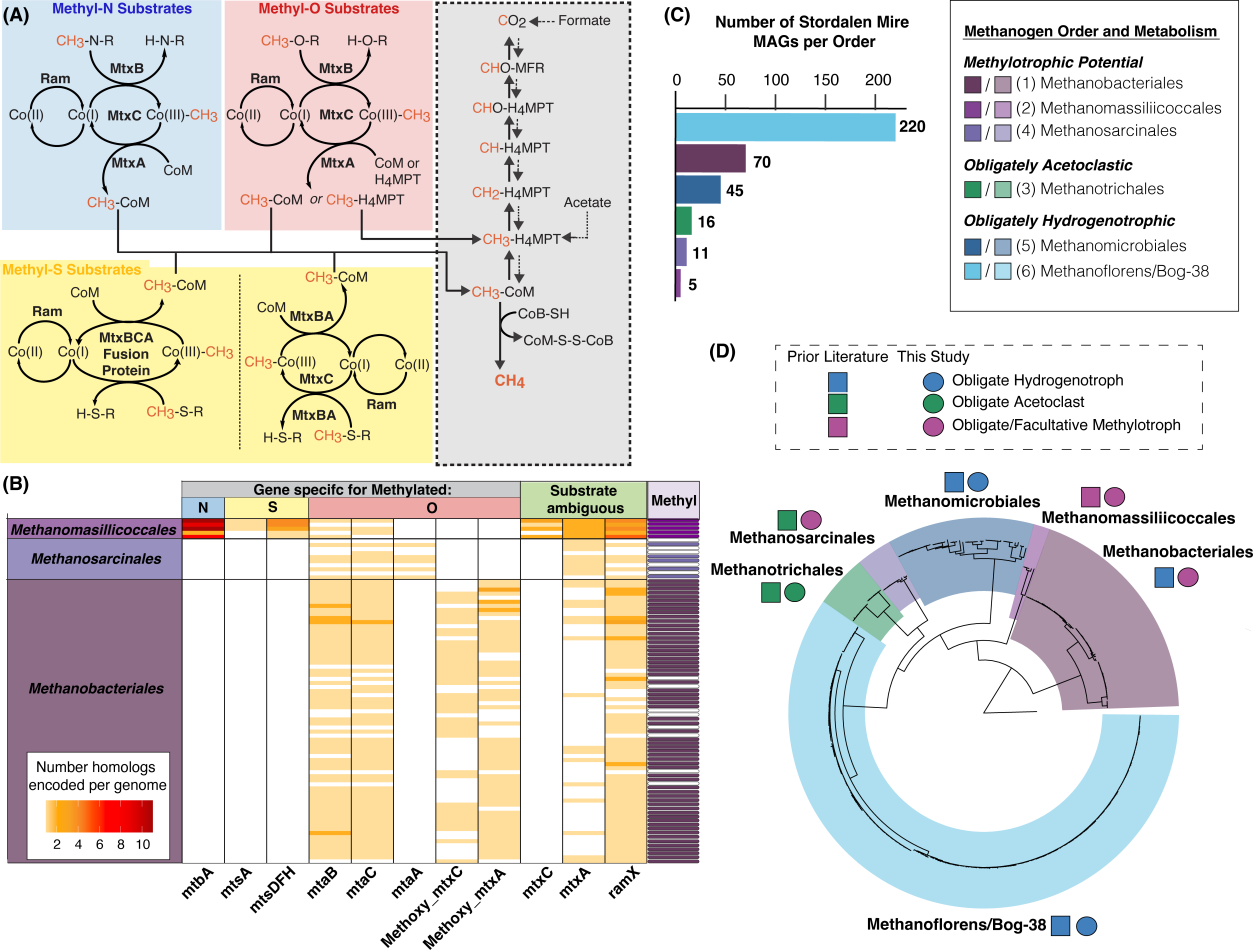
Phylogenomic and metagenomic analyses of Stordalen Mire methanogens. (**A**) Overview of substrate-specific physiology of methylotrophy, involving three-component (or corrinoid-dependent) methyltransferase systems to shuttle substrate-derived methyl groups into central methanogenesis (shown in gray). This includes a substrate:corrinoid methyltransferase (MtxB), a corrinoid-binding protein (MtxC), a methylated-corrinoid:carbon carrier methyltransferase (MtxA), and an activating enzyme (Ram or RamX). The internal “x” in the MtxABC protein/gene name (and the terminal “X” in RamX) is a generalized placeholder; the actual letter at this position varies to denote substrate specificity. Methyltransferase systems for methylated amines and oxygen compounds share a conserved architecture, while those for methylated sulfur compounds occur in two variations involving multi-functional proteins. (**B**) Heatmap showing the number of identified methylotrophic genes encoded in putative methylotrophic methanogen metagenome-assembled genomes (MAGs) from three orders. Each row represents a distinct MAG (grouped by taxonomic order), each column represents a different methylotrophic gene type, and cell color intensity denotes the number of identified genes. Gene columns are grouped by inferred substrate categories: methyl-N, methyl-O, and methyl-S, as in panel **A**, and “substrate ambiguous” for those with annotation uncertainty or known substrate flexibility. Genes only identified in the overall substrate category retain “x” in their names, and in the methyl-O substrate category, methoxylated substrates are indicated by the “methoxy” prefix. For more confident identification of methylotrophic gene homologs, columns are named as substrate-specific methyltransferase system member genes (e.g., the methanol-specific *mtaB*). The furthest right column indicates whether each MAG meets the threshold criteria to be defined as methylotrophic (purple cell) or not (white cell). (**C**) Bar chart showing the number of Stordalen Mire-derived methanogen MAGs per order present in the data set. (**D**) Overlay of phylogeny and genomically inferred function for these 367 methanogen MAGs. MAGs were placed onto the GTDB r207 tree using 53 concatenated archaeal marker genes, and the tree was rooted with a GTDB-derived MAG from the archaeal phylum *Undinarchaeota*. Methanogen orders are delineated by color shading of the tree, and adjacent to each, the genome-inferred methanogen pathway for the representatives at Stordalen Mire is denoted by colored squares for past metabolic designation and circles for this study’s updated designation.

To further assess methylotrophic potential, we manually inspected MAGs for genes encoding three-component (or corrinoid-dependent) methyltransferase systems (Fig. S3A; Table S2 tabs 4–9), each composed of a substrate:corrinoid methyltransferase (MtxB), a corrinoid-binding protein (MtxC), and a methylcorrinoid:carbon-carrier methyltransferase (MtxA), which together bring substrate-derived methyl groups into methanogenesis, and a reductive activase (RamX), which reactivates the corrinoid (Fig. 1A). MAGs were analyzed considering both methyltransferase system component completeness and gene synteny, as *mtxBCA*/*ramX* genes are frequently co-encoded (30, 38, 49). This led us to identify 85 MAGs encoding a total of 438 methyltransferase system genes (Fig. 1B; Table S2 tab Gene_ID_per_trees). These methyltransferase system genes were encoded in multiple representatives within the methanogenic archaeal orders, including all 5 MAGs within the *Methanomassiliicoccales*, all 70 MAGs within the *Methanobacteriales*, and within 10 (of 11) MAGs in the *Methanosarcinales*. In addition, we required a substrate-specific *mtxB* gene in all methanogenic MAGs considered methylotrophic, as we considered this to be the best single marker gene for physiology, due to its encoding of the enzyme that directly catalyzes substrate demethylation. This more conservative requirement retained all of the *Methanomassiliicoccales*, 62 of the *Methanobacteriales,* and 5 of the *Methanosarcinales*. From the genome potential, we reported that the *Methanosarcinales* and *Methanobacteriales* are likely facultative methylotrophs, and the *Methanomassiliicoccales* are likely obligate methylotrophs (Table S2 tab MAG_Physiology_Summary). In summary, we conservatively identified 72 methanogen MAGs with the potential to catalyze methylotrophy (Fig. 1B).

The *Methanomassiliicoccales* MAGs were found to encode genes for diverse methylotrophic substrates, including methylated amines, methylated sulfides, and methanol (Fig. 1B; Table S2 tab Gene_ID_per_trees). The *Methanobacteriales* and *Methanosarcinales* encoded genes solely for the demethylation of methylated oxygen compounds (e.g., methoxylated compounds or methanol), especially homologs of the methanol methyltransferase system components (Fig. 1B). Though these latter two orders were previously considered hydrogenotrophic and acetoclastic, respectively, within Stordalen Mire (41, 42), they each include methylotrophic isolates, providing support for our genomic inferences (24, 50–52). Our metabolic curation of the remainder of the methanogenic orders present in the site (Fig. 1C and D) was in agreement with prior metabolic assignments (41, 42). In total, these data refute the notion that the *Methanomassiliicoccales* are the only lineage within the Stordalen Mire methanogen community to encode the potential for methylotrophic methanogenesis, highlighting the underappreciated potential of this metabolism.

### Methanogen relative abundance and diversity increased along the permafrost thaw gradient

**Fig 2:**
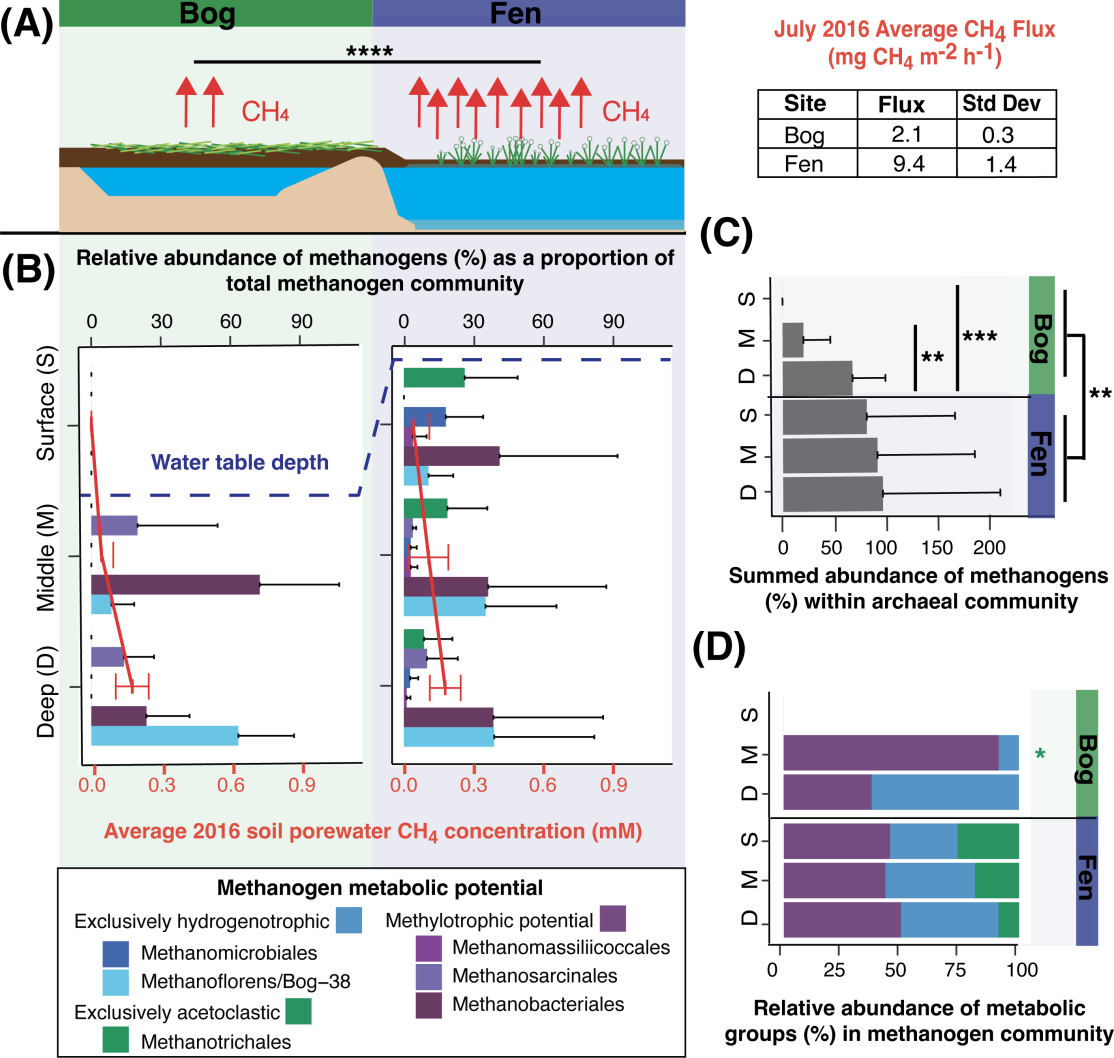
Methanogen abundance and methane flux increased significantly across the Stordalen Mire permafrost thaw gradient in July 2016. (**A**) Cartoon showing the structure of the methanogenic habitats in Stordalen Mire. Red arrows represent the average methane flux from July 2016, with actual values shown in the table on the right. Flux from the fen is significantly higher than from the bog as per Tukey’s Honest Significant Difference (HSD) (*P*-adjusted < 0.0001). (**B**) Bar chart showing the site- and depth-stratified metagenome-based relative abundance of methanogenic orders within the methanogen community, colored by inferred metabolic potential. The dashed line represents the average water table depth at the time of field sampling. The overlayed red line plot shows the soil porewater methane concentrations from July 2016. Error bars represent one standard deviation for both plot types. The palsa habitat (not shown) showed near-negligible production, with values for methanogen relative abundance below detection. (**C**) Summed relative abundance of all methanogens within the archaeal fraction of the soil microbiota. Error bars represent one standard deviation (and the *x*-axis extends beyond 100% due to error bars). Significant differences were seen within the bog via Tukey’s HSD between the middle and deep (*P*-adjusted < 0.01) and the surface and deep (*P*-adjusted < 0.001). Further, a significant difference in the overall site abundance of methanogens between the fen and the bog was found (*P*-adjusted < 0.01). (**D**) Summed relative abundance of metabolic groups (methylotrophic orders, hydrogenotrophic orders, and acetoclastic orders) within the methanogen community. The relative abundance of acetoclastic methanogens was significantly lower than that of methylotrophs in the bog at middle depth (Tukey’s HSD, *P*-adjusted < 0.05); otherwise, no significant differences in the abundance of acetoclasts or hydrogenotrophs relative to methylotrophs were noted.

The Mire contains three distinct habitat types that constitute a discontinuous, natural permafrost thaw gradient. In July 2016, we sampled the active layer in three habitat types: (i) palsa, overlaying intact permafrost, (ii) bog, with partially thawed permafrost and a perched water table, and (iii) fen, fully thawed and inundated (Fig. S1, methods). CH_4_ flux increased with the permafrost thaw state, from negligible from the palsa to highest from the fen (Fig. 2A, Table S3 tab CH4_Flux). Consistent with this flux pattern, we failed to recruit reads to our methanogen MAGs from palsa metagenomes (Table S4 tabs 2–5), while the diversity of the methanogen orders observed here (Fig. 2B) and the total relative abundance of methanogens (Fig. 2C) increased from bog to fen. Additionally, methanogen relative abundance increased significantly with depth in the bog, mirroring the water table depth and likely reflecting saturated anoxic soil conditions favorable for methanogenesis (Fig. 2B). Taken together, our findings support the idea that methanogens in Stordalen Mire chiefly reside in saturated soils.

The relative abundance of methanogenic taxa presented here is in agreement with past studies in regard to overall community composition (42). However, prior research from Stordalen Mire emphasized the primary importance of hydrogenotrophs—largely *Methanoflorens*—in the bog and the appearance of acetoclastic *Methanotrichales* in the fen (41, 42). In this study, our expanded sampling and extended genome-resolved physiological curation of these methanogen MAGs revealed a more metabolically diverse community in both the bog and the fen. Both obligate (*Methanomassiliicoccales*) and assigned facultative (*Methanosarcinales* and *Methanobacteriales*) methylotrophic methanogens were found in both bog and fen habitats. Furthermore, the summed abundance of the hydrogenotrophic orders did not differ significantly from that of the methylotrophic orders in either habitat or depth (Fig. 2D). When considering the presence of individual MAGs within these orders, on average, 64% of the bog (excluding the drained, unsaturated surface) and 30% of the fen methanogen communities encoded the potential for methylotrophic methanogenesis. Our genomic analyses support representation of methylotrophic methanogenesis in this climatically critical ecosystem, warranting further investigation into the chemistry and expressed physiology supporting this metabolism.

### Stordalen Mire peat contains methylotrophic substrates, especially methanol

**Fig 3:**
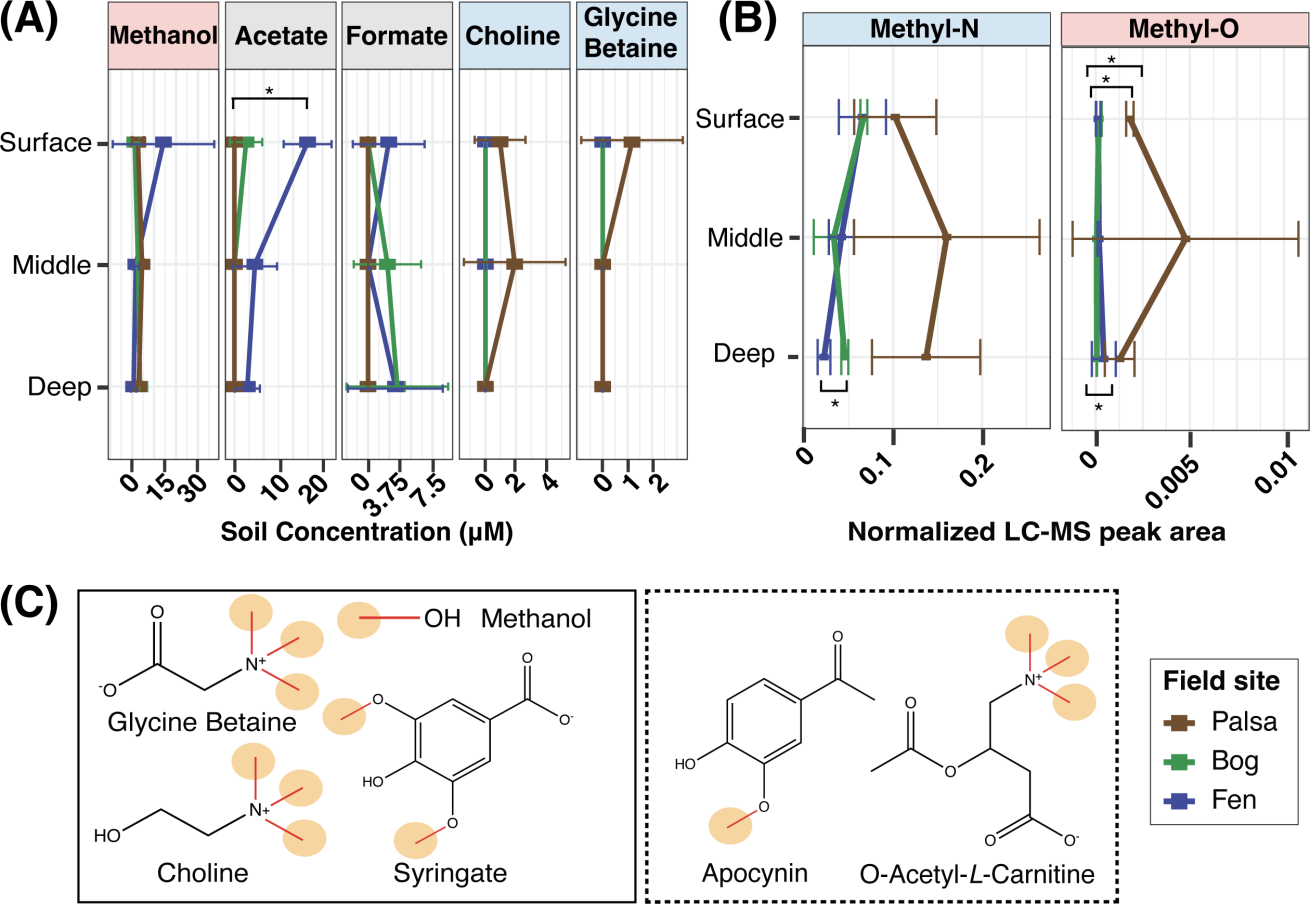
Diverse methylated metabolites are present across Stordalen Mire. (**A**) Known methanogenic precursors found in peat water extracts as detected by NMR. Points represent average concentrations, with error bars representing one standard deviation. Significant differences were assessed using Tukey’s HSD, with only acetate found to be significantly enriched in the fen compared to the palsa (*P*-adjusted < 0.05). (**B**) The summed LC-MS/MS peak area for five methylated amines and four methylated oxygen compounds across habitat and depth, which are implicated as potential methylotrophic substrates (Table S3; Fig. S3). Methylated amines were found to be significantly higher in the fen than in the bog (Tukey’s HSD, *P*-adjusted < 0.05), and a significant difference was observed for methylated oxygen compounds between each pair of sites via Tukey’s HSD (fen:bog, *P*-adjusted < 0.05; palsa:fen, *P*-adjusted < 0.05; palsa:bog, *P*-adjusted < 0.05). (**C**) Chemical structures of select known (solid box) and here proposed possible (dashed box) substrates identified in Stordalen Mire, with microbially available methyl groups circled in orange.

To further characterize the likelihood of methylotrophy, we next analyzed peat water extracts to detect possible methanogenic substrates. Quantitative nuclear magnetic resonance (NMR) analysis identified 29 such metabolites (Table S3 tab NMR_data), including classical methanogenic substrates like acetate and formate, as well as the methylated oxygen compound methanol (Fig. 3A). The methylated amines glycine betaine and choline were also detected but only in the non-methanogenic palsa (Fig. 3A). Liquid chromatography tandem mass spectrometry (LC-MS/MS) also identified methylated compounds as present across habitats (Table S3 tabs 7–9; Fig. S3B and C), including four methylated amines and four methylated oxygen compounds (Fig. 3B). Of these compounds, only three (glycine betaine, choline, and syringate) are recognized as known substrates for this metabolism. Most of the remainder are small derivatives of known substrates (e.g., acylated methylated amines or their stereoisomers) or chemical species with structural homology to known substrates (e.g., apocynin). Notably, trigonelline is chemically distinct and could represent an as-yet unknown aromatic methylamine to support this metabolism.

A relevant consideration when trying to link methylotrophic substrates to ecosystem outputs like CH_4_ is that the number of potentially microbially available methyl groups on different compounds (Fig. 3C) may limit the stoichiometry of CH_4_ formation (18, 20, 21, 38). For example, one molar equivalent of a tri-methoxylated compound may support the production of three times as much CH_4_ as one molar equivalent of methanol. However, the broad availability of methanol found across the Mire (e.g., its detection in each palsa, bog, and fen), which may be ultimately plant derived and therefore continually produced in the site (53), led us to postulate that methanol might be a primary substrate for methylotrophic methanogenesis *in situ*. In support of this, 83% of all methanogen MAGs classified here as methylotrophic were identified as encoding the gene for the methanol-specific methyltransferase MtaB (Fig. 1B).

### Methanogens express methylotrophic genes across the Mire

**Fig 4:**
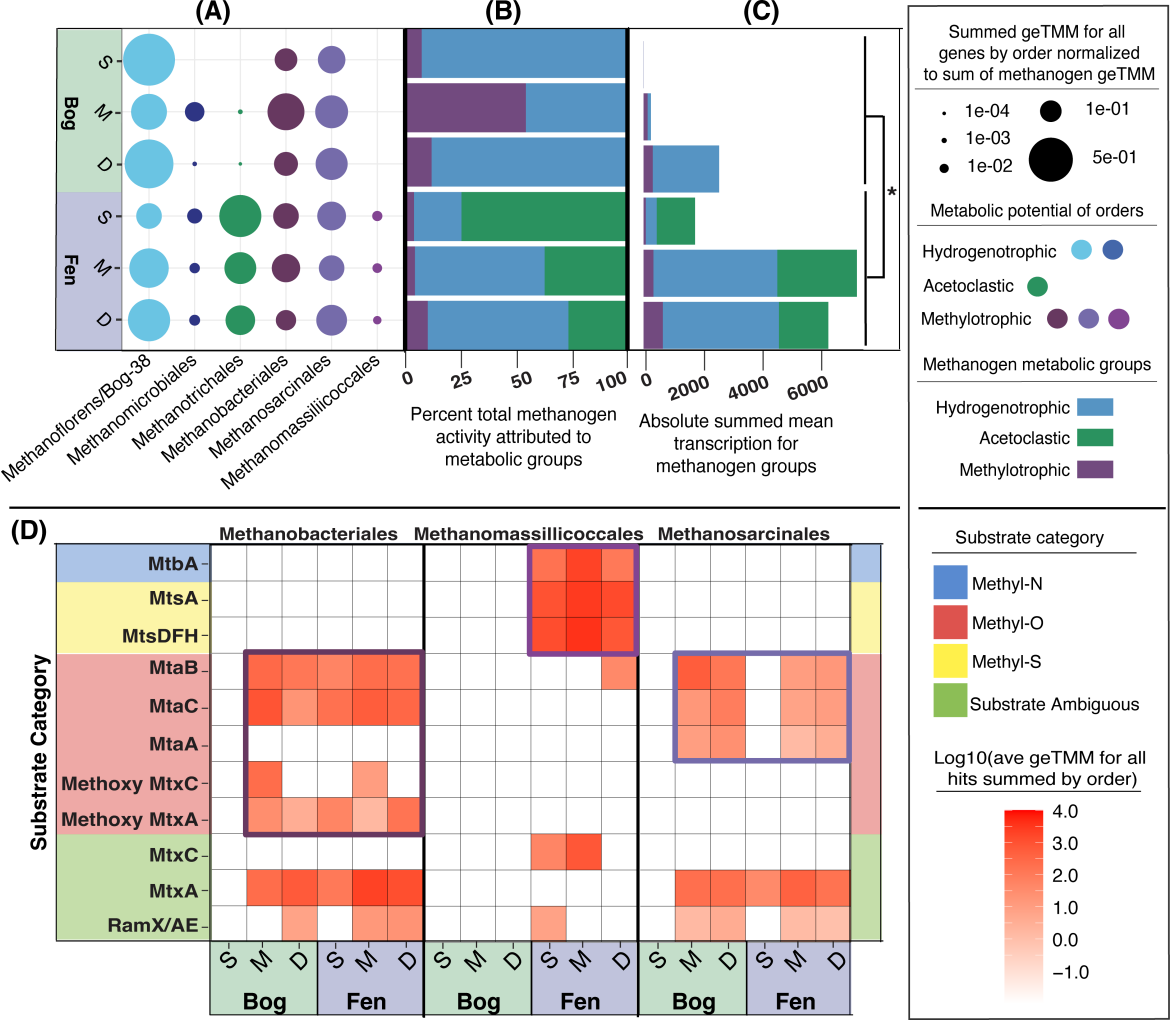
Methanogens with methylotrophic potential are active and expressing genes for methylotrophy across the Mire. (**A**) Summed relative transcriptional activity of methanogen orders across the Mire within the methanogen community, calculated as averaged geTMM values for all methanogen-expressed genes summed at the order level and normalized to the total sum of all methanogen-expressed genes. (**B**) Bar chart showing the percent of the total methanogen activity at each depth within the bog and the fen attributable to metabolic groups of hydrogenotrophs, acetoclasts, and methylotrophs. (**C**) Bar chart showing the absolute summed mean transcription of methanogen metabolic groups across the bog and fen. Total transcription is significantly higher in the fen than in the bog (Tukey’s HSD, *P*-adjusted < 0.05), but no significant intra-habitat differences were seen between the activity of individual metabolic groups. (**D**) Specific expression of methylotrophic genes by three methanogenic orders across the Mire. geTMM values for expressed methylotrophic methyltransferase genes averaged and normalized to MAG relative abundance within metatranscriptomes and plotted across depth profiles within the bog and fen. Expressed genes are categorized by inferred substrate category. Purple boxes are used to highlight the apparent primary substrate-specific genes expressed by each order. Evidence for active methylotrophic methanogenesis is presented across the bog and fen at every depth except the bog surface (which is likely the most oxygenated field compartment of the six represented here).

Following investigation of the genomic and chemical potential for methylotrophic methanogenesis, we queried the expression of the putative methylotrophs using genome-resolved metatranscriptomics (Tables S1 tab MetaT_Accession_Info and S2 tabs 10–11). Here, we show that all three potentially methylotrophic orders are active *in situ* across habitats and depths (Fig. 4A). Gene-resolved expression analysis confirmed that almost all (70%) of the active MAGs in these orders were expressing methylotrophic genes (Fig. S4). Notably, these actively methylotrophic MAGs represent on average 100% of the summed activity of the *Methanomassiliicoccales*, 85% of the activity of the *Methanosarcinales*, and 91% of the activity of the *Methanobacteriales* across the bog and fen (Fig. S4).

In total, methylotrophic orders accounted for between 5% and 10% of the total methanogen transcription in the fen and a broader 7%–54% in the bog (Fig. 4B), with the greatest proportion of active methylotrophic orders observed in the bog at middle depth. Consistent with our CH_4_ flux data, overall methanogen activity increased significantly between the bog and the fen (Fig. 4C). Unlike the acetoclastic methanogens, which had significantly increased activity in the fen, no such significant habitat pattern was seen for the methylotrophs. However, per the increased total methanogen activity in the fen relative to the bog, the absolute activity of methylotrophs in the fen, though a smaller average proportion of the methanogen community than in the bog, may not represent an actual decrease in activity with thaw (Fig. 4C). Our findings indicate that methylotrophic methanogens play an active role within the Stordalen methanogen community and thus contribute to the CH_4_ cycle *in situ*.

Our gene expression data were used to refine the substrate usage patterns for these methylotrophic lineages (Fig. 4D). For the *Methanomassiliicoccales*, our metatranscript data suggest that methylated sulfides, and possibly methylated amines, are more likely substrates than methylated oxygen substrates due to the limited expression of methanol or methoxy genes. On the other hand, the facultative methylotrophs *Methanosarcinales* and *Methanobacteriales* exclusively had the potential for methylated oxygen usage. Gene expression data supported the use of methylated oxygen compounds, especially methanol, across the bog and fen by these two lineages. It should be noted however that members of all three orders were found to express substrate ambiguous methylotrophic genes, which are not used here to assign functional substrate profiles.

Taken together, our combined metagenomic, metatranscriptomic, and metabolomic data demonstrate active methylotrophic methanogenesis across Stordalen Mire using field-relevant substrates by a sizeable fraction of the native methanogen community. Our expression data also hint at methylotrophic niche partitioning that may occur in the fen, with the *Methanomassiliicoccales* showing a preference for methylated sulfide substrates and the *Methanosarcinales* and *Methanobacteriales* preferentially utilizing methylated oxygen substrates. This simultaneously improves our understanding of the CH_4_ cycle in this climate-critical wetland and demonstrates the need for genome-resolved approaches to studying methanogen physiology.

### Anaerobic methylotrophy is encoded and expressed by numerous bacteria in Stordalen Mire

**Fig 5:**
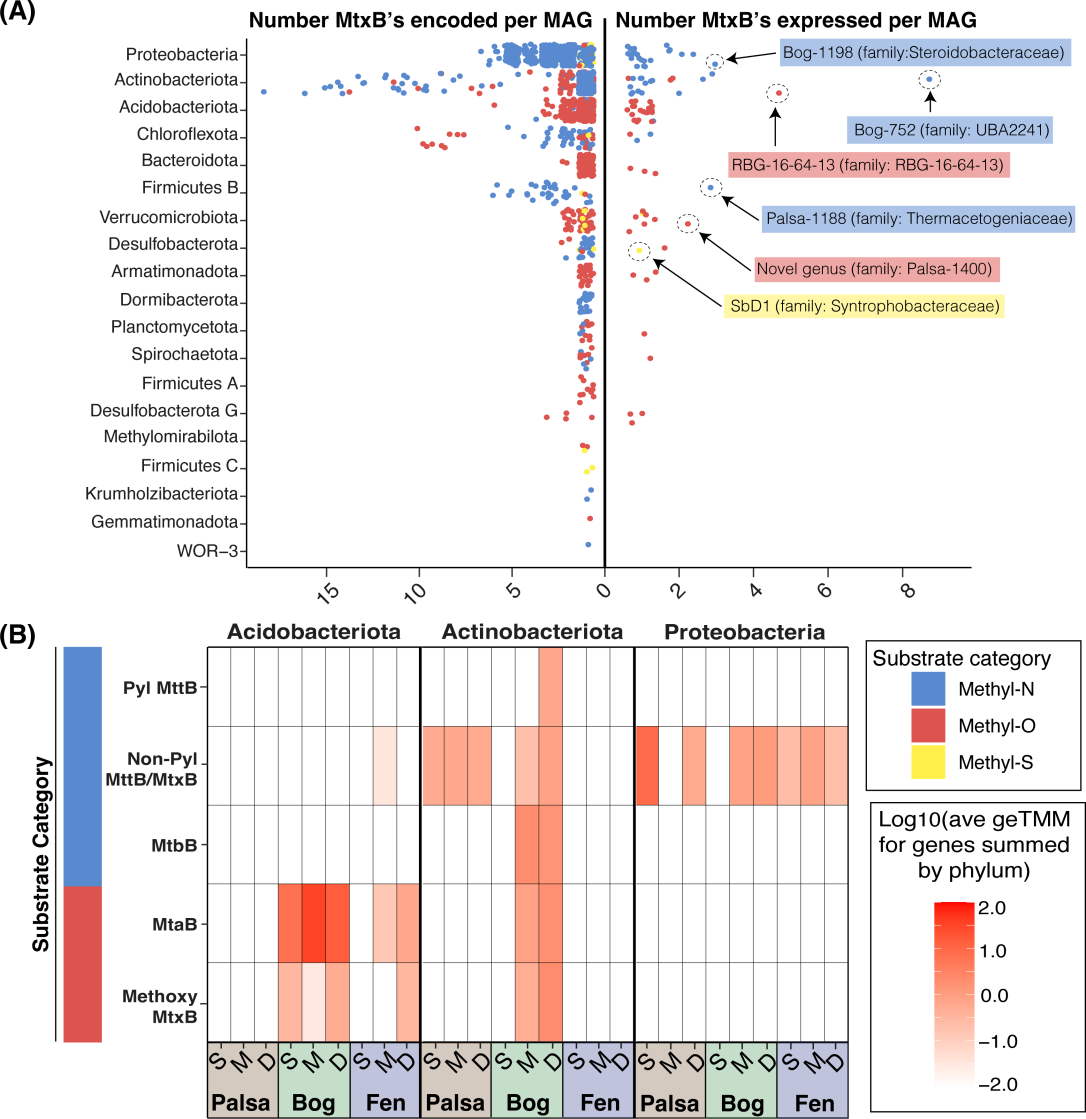
Anaerobic methylotrophic metabolism extends to the bacterial component of the soil microbiota in Stordalen Mire. (**A**) Plots showing the number of *mtxB* genes encoded (left) and expressed (right) per MAG within bacterial phyla, colored by inferred substrate specificity, in Stordalen Mire. Approximately 1,700 bacterial MAGs spanning 19 phyla encode *mtxB* genes, of which 88 from 12 distinct phyla were found to be actively expressing these genes. The genus (and family) of some active methylotrophic bacteria is shown on the right plot to demonstrate the here-observed taxonomic diversity of the metabolism in Stordalen. (**B**) Specific expression of identified *mtxB* genes by the three phyla found to include the greatest number of putatively methylotrophic bacteria in the Mire. Bacterial methylotrophic gene expression is evident across the entirety of Stordalen Mire in both the methanogenic and non-methanogenic habitats.

Some anaerobic bacteria employ homologs to the methanogenic three-component methyltransferase systems, where these same methylotrophic substrates support growth as sources of carbon and/or energy (30, 31, 33–38). Investigation of the methylotrophic potential among the so-far identified bacterial component of the soil microbiota in Stordalen revealed that >1,700 MAGs from 19 bacterial phyla encoded thousands of *mtxB* genes (Fig. 5A; Table S5 tab BLAST_bitscore > 200). These genes were predominantly inferred to be specific for methylated amines, methanol, and methoxylated compounds. However, some MAGs encoded homologs of methylated sulfide-dependent methyltransferases (Fig. 5A), representing to our knowledge the first environmental identification of bacterial methylated sulfide methyltransferase systems. Overall, these data are in good agreement with, and expand upon, the findings of Ticak et al. (30) and Creighbaum et al. (20) on the broad phylogenetic diversity of methylamine-dependent *mtxB* genes extending past solely methanogenic archaea and acetogenic bacteria.

Of the 19 bacterial phyla found here to encode *mtxB* genes, members of 12 phyla were found to express these genes (Fig. 5A), suggesting active bacterial methylotrophy within the Mire. The *Proteobacteria*, *Acidobacteriota*, and *Actinobacteriota* appeared to be the primary methylotrophic bacterial phyla in the site (Fig. 5A and B), with members of genera *Bog-752*, *RBG-16-64-13*, and *Bog-1198* being their most actively methylotrophic representatives. The *Acidobacteriota* exclusively expressed genes for the demethylation of methanol (*mtaB*) and methoxylated compounds across the bog and fen. Members of the *Proteobacteria* expressed *mttB* homologs lacking the unique pyrrolysine residue (non-Pyl), which are known to be specific for quaternary methylated amines such as choline and glycine betaine (30). Notably, members of this phylum expressed these genes in the palsa, where NMR revealed the presence of these compounds (Fig. 3A) and where our multi-omics failed to detect the presence or activity of methanogens. Last, the *Actinobacteria* were the most versatile, with expression of genes for the demethylation of both methylated amines and methylated oxygen compounds. Interestingly, some MAGs within the *Actinobacteriota* were found to express pyrrolysine-encoding *mttB* and *mtbB* genes, which are specific for tri- and dimethylamine, respectively (54). Our findings illustrate new insights into the bacterial transformation of methylated substrates, both phylogenetically showing the activity of diverse lineages and also indicating new substrates for bacterial methylotrophs.

## DISCUSSION

**Fig 6:**
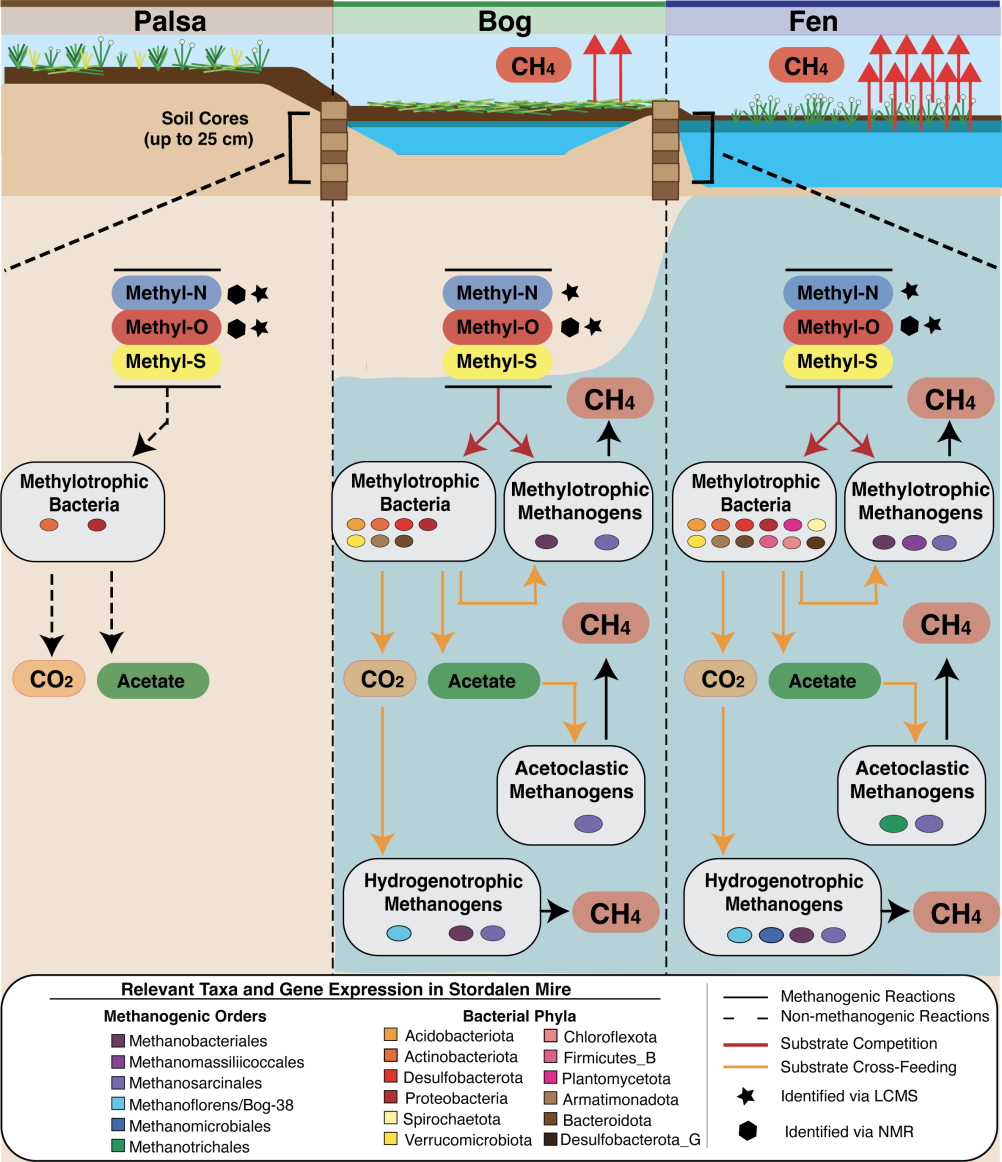
The anaerobic methylotrophic network in Stordalen Mire. Metatranscriptome-informed conceptual model summarizing the complexity of the microbial anaerobic methylotrophic food web in Stordalen Mire. Vertical dashed lines separate the palsa, bog, and fen, and the blue background is intended to represent the water table depth within sampled soil cores across habitats relative to microbial metabolic activity. Solid arrows represent metabolic reactions that can lead to the production of CH_4_ either directly or indirectly, while dashed arrows represent reactions not expected to result in CH_4_ production. Red arrows reflect substrate competition, while yellow arrows reflect cross-feeding of different metabolic groups. All represented bacterial taxa in the figure include MAGs found to express methylotrophic methyltransferase genes (*mtxB*) in Stordalen Mire. Substrate categories identified within each habitat per metabolite data are represented by stars (LCMS-identified) and hexagons (NMR-identified). Note that the metabolic versatility of the facultatively methylotrophic *Methanosarcinales* and *Methanobacteriales* is represented by their dual inclusion in multiple methanogenic metabolic groups.

The aim of this study was to employ a genome-resolved approach to query the potential for methane-relevant anaerobic methylotrophy, catalyzed by corrinoid-dependent methyltransferase systems, among the microbiota in a thawing permafrost peatland. Here, paired metagenomic and metatranscriptomic data in conjunction with metabolite analyses revealed substantial evidence for the metabolism of methylated compounds by members of the soil microbiota (Fig. 6). We classified nearly 20% of all known Stordalen-derived methanogen MAGs as methylotrophic and further supported their activity in saturated soils in the bog and fen using integrated metabolite and metatranscriptomic evidence. Moreover, we extended the role of methylotrophy to the bacterial members of the community, providing a framework for how this metabolism could further compete with or support methanogenesis in a more indirect fashion (Fig. 6). Beyond Stordalen, our field data may reciprocally broaden knowledge of the metabolism itself with the identification of wetland-relevant potential novel substrates, plus underappreciated microbial cross-feeding and competition, explored below, which warrant further physiological experimentation.

### Methylotrophic methanogenesis is encoded by much of the native methanogen community

Though the historical paradigm of environmental methanogenesis has suggested methylotrophy to be a niche metabolism of limited relevance across terrestrial wetlands (55–59), recent studies have countered this idea, increasing both the taxonomic (9–13, 60, 61) and geographic (14–17) footprints of the metabolism. However, a notable challenge remains in profiling environmental methylotrophy. Namely, biochemical discoveries of known relevant genes and substrates are not translated accurately to databases used to functionally annotate genomes or metagenomes (e.g., KEGG). For example, four functionally unique quaternary amine-dependent methyltransferases have been biochemically characterized and are shown to demethylate distinct substrates (30, 33, 35, 38), but only one of these is present in the KEGG database (30). As a result, the accurate functional assignment of these genes often requires paired non-homology-based methods, including gene synteny (19, 30, 33, 38, 62), unique residues (54, 62), and phylogeny (62) to confirm gene functional assignments.

Consequently, holistic surveys of environmental methanogenesis that lack manual methylotrophic curation only screen for a limited number of best-known genes and thus likely underestimate the overall potential for and diversity of the metabolism. Our annotation strategy to profile methylotrophy in Stordalen Mire did not rely solely on annotations from these databases. For example, via database annotations only, we would have missed most of the *Methanomassiliicoccales*-encoded methylated sulfur use potential, as well as the potential for methanogenesis from methoxylated substrates from any lineage.

Of the methanogenic orders identified here as having the potential to catalyze methylotrophy, only the *Methanomassiliicoccales* appear to be functioning as obligate methylotrophs, likely using these substrates dependent on hydrogen as an electron donor (7). Studies have demonstrated that these methanogens are cosmopolitan across water-inundated soils and sediments (14, 63–65). In contrast to prior metagenomic studies (14, 63), we did not infer that methanol was the preferential substrate for this lineage. Instead, our metatranscriptomic findings indicated the preferential utilization of methylated sulfides. While some representative *Methanomassiliicoccales* isolates have been shown to encode dimethyl sulfide-specific *mtsAB* methyltransferase genes (7, 66), no isolates to our knowledge have been shown to grow with methyl sulfides directly (7). Markedly, the Stordalen MAGs were found to encode not only the dimethyl sulfide-specific *mtsA* but also the homologs of tri-functional *mtsD/mtsF/mtsH* genes (23). These genes may confer the ability to use a broader suite of methylated sulfide substrates than *mtsAB* alone, which would expand the physiological potential of the order, as well as the knowledge of its metabolic niche within wetlands.

Beyond the *Methanomassiliicoccales*, we expanded the observation of methylotrophy within this site to include members of the *Methanosarcinales* and *Methanobacteriales*, identifying these lineages as primarily methanol-utilizing facultative methylotrophs. Both orders have been identified as members of methanogenic communities across global wetlands (65, 67–69). However, the *Methanobacteriales* are typically classified solely as hydrogenotrophs (65, 68), whereas the *Methanosarcinales* are inconsistently labeled as acetoclasts (67), acetoclastic and hydrogenotrophic (65), or metabolically versatile (e.g., capable of all modes of methanogenesis) (14, 68). Prior, two *Methanobacterium* isolates (24, 51)—one of which is from a permafrost system (51)—have been shown to perform hydrogen-dependent methylotrophy using methanol and/or methylamines. Meanwhile, the *Methanosarcinales* are known via biochemical and physiological work to perform all modes of methanogenesis, including methylotrophy from diverse substrates such as methanol (23, 50, 52). Vanwonterghem et al. (9) demonstrated methylotrophic potential for numerous genomes within both orders, including methanol use potential among other wetland- (70, 71) and permafrost-relevant members (72, 73). Thus, while both orders are known residents of wetlands, we propose that their classical niches underrepresent their true physiological potential across wetlands, as per their observed gene expression in Stordalen and potential isolation from other sites.

Considering especially the here-identified role for facultative methylotrophs within Stordalen Mire, experimentation is needed to resolve the question of the comparative kinetics of—and thus overall production from—discrete pathways of methanogenesis. This analysis is relevant not only at the methanogen community level but also at the level of a single organism like members of the *Methanosarcina*, where we observed co-expression of acetoclastic and methylotrophic genes in the bog from a single methanogen genome (Table S2 tab Methanogen_geTMM_all_genes). Still, the data presented here imply a more important role for methylotrophy in Stordalen Mire than previously understood. Of the 36 methanogen MAGs identified as being active in this data set, 10 expressed genes for methylotrophy, and we confirmed the presence of metabolites to support some of these dominant pathways (Fig. 6). Regardless of the kinetics, the widespread footprint of this metabolism across the methanogen community precludes dismissal of its consideration at the ecosystem level.

### Bacterial methylotrophy is a cryptic part of the carbon cycle in wetlands

Though an important role is supported here for methylotrophic methanogens in Stordalen Mire, the metabolism is not limited to the archaeal community. From the literature, it is recognized that anaerobic bacteria, especially certain acetogens, perform methylotrophy via corrinoid-dependent methyltransferase systems feeding into the Wood-Ljungdahl pathway (30, 31, 33–38). Here, we observed the expression of *mtxB* genes by bacteria across the entirety of the Mire at nearly all depths, including the drier and undoubtably more oxygenated palsa. While these genes are inferred to be involved exclusively in an obligately anaerobic metabolism, active anaerobes have been identified in oxygenated surface habitats in other locations (74, 75). NMR and metatranscriptomic data support active bacterial methylated amine-dependent methylotrophy in the palsa. While not likely supporting methanogens in this habitat, this bacterial metabolism could be a source of CO_2_ (30), an important greenhouse gas.

Overall, these data suggest that bacterial methylotrophy is active across the Mire. In the CH_4_-emitting bog and fen, we have considered the ways bacterial methylotrophy could impact the CH_4_ cycle. For instance, while bacterial methylotrophy does not directly produce CH_4_, it may produce acetate (30, 33, 35, 35) and CO_2_ (30), which are substrates for acetoclastic and hydrogenotrophic methanogens, respectively. It is also possible that methylotrophic bacteria could cleave quaternary methylated amines (e.g., choline) into smaller methylated amines (trimethylamine) to fuel methylotrophic methanogenesis (76, 77). Methylotrophic bacteria are active in the same habitats and depths as the three metabolic groups of methanogens (Fig. S5A), and notably, a strong positive correlation was identified between the summed transcriptional activity of methylotrophic methanogens and methylotrophic bacteria in the fen (*r*^2^ = 0.87) (Fig. S5B). Alternatively, bacterial methylotrophs could also compete with methanogens for methylotrophic substrates, complicating this understudied component of the microbial carbon food web. For example, our gene expression data indicate that the *Methanosarcinales* and *Methanobacteriales* (Fig. 4D) compete for methanol with the *Acidobacteriota* (Fig. 5B). Taken in total, the apparent complexity of the methylotrophic metabolic network in wetlands warrants future experimental work to better resolve the relevance of these types of metabolism to wetland methanogenesis and the terrestrial global carbon cycle. While our data are specific to Stordalen Mire, we can envision extending this model across wetlands per the growing recognition of the importance of methylotrophy across habitats (14–16).

### Conclusions and future needs

Our study advances the growing recognition of the complexity and ecological relevance of methylotrophy and highlights the power of large-scale field data sets to illuminate its biochemical diversity, phylogenetic extent, and ecological drivers. The methylotrophic metabolic network is increasingly implicated—here and in other climate-relevant ecosystems (14–17)—in impacting atmospheric CH_4_ emissions, especially in a warming climate (17, 78). This expanded knowledge of a widespread metabolism contributing to CH_4_ dynamics in wetlands is essential to improve model-based predictions of wetland contributions to the global CH_4_ budget (79). Process-scale biogeochemical models [like *ecosys* (8)] do not currently account for methylotrophic methanogenesis, representing only acetoclastic and hydrogenotrophic pathways (8, 80)—a likely consequential misrepresentation since methylotrophy dramatically increases the potential route of fixed carbon to CH_4_ production, and its enzymes have distinct kinetics and constraints. This work also provides the field-relevant targets for *in vitro* studies of methyltransferase systems, which are needed to determine field-relevant key kinetic parameters, especially since the so-far kinetically characterized methylotrophic methyltransferases do not well represent known wetland methanogen communities. Quantifying the methylotrophic contribution of methanogenesis in wetlands—the largest natural biogenic CH_4_ source (81)—likely via isotopically labeled substrate experiments (82) is an essential next step in constraining its addition to predictive models. Together, these integrated approaches will increase biological realism in models and predictions for these and other major CH_4_-emitting climate-sensitive habitats.

## MATERIALS AND METHODS

### Field site and sample collection

Stordalen Mire (68 22′N, 19 03′E) is a rapidly thawing Artic permafrost peatland near Abisko, Sweden. The mosaic of permafrost land cover includes three primary biologically and chemically distinct habitats that constitute a discontinuous permafrost thaw gradient. The raised, well-drained palsa overlays intact permafrost with an active layer depth of 50–60 cm and is dominated by woody and ericaceous shrubs. The bog site, dominated by *Sphagnum* mosses, is underlain by partially thawed permafrost and a seasonally fluctuating perched water table. Last, the sedge-dominated fen represents fully thawed and saturated permafrost. In July 2016, cores were taken for meta-omic and geochemical analyses in triplicate using an 11-cm-diameter push corer from each palsa, bog, and fen in areas adjacent to the *in situ* gas flux measurement autochamber system. Cores were subsectioned in the field at three depths: surface (1–4 cm), middle (10–14 cm), and deep (20–24 cm). Subsections were split based on end-use; for nucleic acid extraction, 4 mL of the field-saturated peat was added to 2.5 volumes of Lifeguard buffer (Qiagen, MD, USA), transferred out of the field on ice in a cooler, and frozen at −80°C until extraction. A second split—a portion of which was used for metabolomics—was placed in a 50-mL Falcon tube without buffer, flash frozen in liquid nitrogen, transferred out of the field on ice in a cooler, and stored at −80°C until processing.

### Methane field measurements

To determine soil porewater CH_4_ concentrations, prior to coring at each site, porewater was collected with a perforated stainless-steel piezometer inserted into the peat and extracted with an airtight syringe. No porewater was obtained from sites (palsa) or depths (in the bog or fen) that were above the water table. Porewater samples were filtered and acidified and stored in evacuated vials until they were brought to atmospheric pressure with helium, and the CH_4_ concentration in the equilibrated headspace was measured using a flame ionization detector gas chromatograph. An extraction efficiency of 0.95 was used to calculate the dissolved CH_4_ concentration.

CH_4_ fluxes were measured using a system of eight automated gas-sampling chambers made of transparent Lexan (*n* = 3 each in the palsa, bog, and fen habitats, with *n* = 2 in the fen prior to 2011). Chambers were initially installed in 2001 (83), and the chamber lids were replaced in 2011 with the larger current design, similar to that described by Bubier et al. (84). Each chamber covers an area of 0.2 m^2^ (45 cm × 45 cm), with a height ranging from 15 to 75 cm depending on habitat vegetation. At the palsa and bog sites, the chamber base is flushed with the ground, and the chamber lid (15 cm in height) lifts clear off the base between closures. At the fen site, the chamber base is raised 50–60 cm on Lexan skirts to accommodate large-stature vegetation. The chambers are instrumented with thermocouples measuring air and surface ground temperature, and water table depth and thaw depth are measured manually 3–5 times per week. The chambers are connected to the gas analysis system, located in an adjacent temperature-controlled cabin, by 3/8″ Dekoron tubing through which air is circulated at approximately 2.5 L min^−1^. Each chamber lid is closed once every 3 h for a period of 8 min, with a 5-min flush period before and after lid closure. The results for multiple years are reported by Holmes et al. (40).

The July 2016 CH_4_ flux data and porewater data are in Table S3 tabs 4–5; the data for field sites, including porewater CH_4_ and water table depth, can be found at 10.5281/zenodo.7720573, and the July 2016 flux data used in Fig. 1 can be found at 10.5281/zenodo.7897922.

### DNA and RNA extraction

To produce sequence data for previously unpublished MAGs (Table S1 tab MetaG_Accesion_Info), as well as to generate metatranscriptomes for this study (Table S1 tab MetaT_Accession_Info), DNA and RNA were each extracted using the Mobio PowerMax Soil DNA/RNA Isolation Kit (cat# 12966–10). Sample vials were removed from the −80°C freezer and thawed on ice. Following this, 5–10 g of peat material (preserved in Lifeguard soil preservation solution, Qiagen) was added to bead tubes, and nucleic acids were extracted per the manufacturer’s guidelines without the initial addition of beta-mercaptoethanol. Reagents were increased proportionally to maintain the concentration of solutions. An additional ethanol wash of the nucleic acid-bound column was performed to further remove impurities. The resulting washed nucleic acid was eluted with 5 mL of RNase-free DI water and further concentrated via ethanol precipitation overnight, followed by elution in 100 µL of TE buffer. The eluted product was further processed for separation and purification of each DNA and RNA; samples were aliquoted into two 2-mL tubes at a ratio of 1:2. RNase and DNase treatment (Roche) were both performed following the manufacturer’s guidelines, followed by phenol:chloroform purification. Separated nucleic acids were then ethanol precipitated, and pellets were eluted in TE buffer. Purified DNA and RNA were each quantified via Quibit 3.0. All samples were stored at −80°C pending sequencing.

### Metagenome assembly and binning

To maximize the site-specific MAGs available for this study, they were developed from three sources: (i) all those generated from Woodcroft et al. (43), (ii) assembly and binning of metagenomes from 2010 to 2017 field samples, and (iii) all fractions of a stable isotope probing experiment performed on the field peat added to labeled plant matter from locally co-occurring species. Stable isotope probing metagenomes were used here purely to add site MAGs, not for analyses of that experiment.

Metagenome read sets from 2010 to 2017 field samples were trimmed using Trimmomatic (v0.36) (85) in the paired-end mode against the TruSeq3-PE 23argaret adapters with a sliding window of 4–15. Trimmed reads were then assembled with SPAdes (v3.12, --meta option enabled) (86) with the default kmer set. Sample 20120700_E3M was too complex to assemble the first kmer set within our computational limits, so the reads were randomly subsampled to 50% using bbtools (v38.51) (87) reformat.sh and assembled with metaSPAdes (88). The contigs from this sample were then dereplicated before subsequent steps with cdhit (v4.8.1) (89) with the following parameter sets: -c .99 -aS .80 -n 11 -d 0 -g 1. Read mapping was done from the quality-controlled reads against all samples. Once assembly and read mapping were complete, the bam files and contigs were used as input for binning. An initial bin set was created using UniteM (v0.0.18) (90) with the following options: mb_sensitive, mb_verysensitive, mb_specific, mb_veryspecific, mb2, max40, max107, bs, and gm2. Next, UniteM, DAS Tool (v1.1.1) (91), and MetaWRAP (v1.0.6) (92) were used to create ensemble bin sets. Due to the limitation of the MetaWRAP bin_refinement module only accepting three candidate bin sets, MetaBAT2 (93), GroopM2 (94), and MaxBin2 (95) from the initial bin sets were used as input into MetaWRAP. The output of these ensemble tools was then used as input into the same tools (DAS Tool, MetaWRAP, and UniteM) for a second iteration of ensemble binning.

Completion and contamination statistics of the second iteration of ensemble bins were determined using the CheckM (v1.0.12) (96) lineage workflow. Bins with at least 70% completion and less than 10% contamination were leveraged to determine a quality score of the three ensemble bin sets for each sample individually. The quality score was calculated as follows: score = completeness – (5 × contamination). The ensemble binning tool with the highest quality score was used as the bin set for that sample. For any samples where the number of bins generated from the first step of UniteM was too large for our computational limits, MetaBAT2 was used alone. Once a candidate bin set was chosen, RefineM (v0.0.24) (97) “outliers” was run using the following parameters: --td_perc 95 –gc_perc 95. All MAGs with at least 70% completion and less than 10% contamination were then manually examined and refined through anvi’o 25argaret (v5.2) (98), leveraging differential coverage and GC content with hierarchical clustering guiding refinements.

Read sets from the SIP experiment were trimmed identically, though they were assembled with both SPAdes (--meta option enabled) (v3.13) with the default kmer set and with MEGAHIT (v1.1.3) (99) with the default kmer set. Additionally, BFC error (100) correction was performed and assembled with SPAdes (v3.13) with the default kmer set. For all samples that had two sets of reads, the largest read set was assembled. Abundance information for each contig was generated using Bowtie2 (v2.4.1) (101) by mapping reads from all samples (without T0), within the same habitat, to all contigs, for each assembly. T0 reads were only used on assemblies generated from T0 samples. Each assembly was binned separately into MAGs using MetaBAT2 (v2.12.1).

### Additional sources

Genomes and assemblies for the stable isotope probing samples were downloaded from JGI on 7 December 2020 and 4 December 2020, respectively. These data sets were generated through the DOE-JGI metagenome annotation pipeline (102).

From these total efforts, a database of 13,290 medium- and high-quality (103) MAGs was generated using data from 882 Stordalen Mire field and microcosm metagenomes spanning sampling from 2011 to 2017. MAGs were annotated using DRAM (v1.3.2) (44). Taxonomy was assigned using GTDB-Tk (v2.1.1 r207) (104). These 13,290 MAGs were dereplicated at 97% identity using dRep (105) into 1,864 representative MAGs with galah (v0.3.0) (106) using the following parameter set: --precluster-ani 90 –ani 97 –precluster-method finch. Accession information for metagenomic reads is provided in Table S1 tab MetaG_Accesion_Info, and the full database of 13,290 MAGs can be downloaded from https://doi.org/10.5281/zenodo.7596016.

### Metatranscriptome analysis

Metatranscriptome libraries were prepared for 27 field samples at the University of Colorado Anschutz Medical Campus. Using 10 ng RNA for each, rRNA was first depleted using the QIAseq FastSelect −5S/16S/23S (Qiagen) kit per the protocol with some modifications to follow the library protocol used by the Joint Genome Institute: probes for both plants and yeast were added, and only one-third of the probe volumes were used. Next, the TruSeq Stranded Library Preparation Kit (Illumina) was used to prepare the sequencing library. Libraries were sequenced on an Illumina NovaSeq 6000 system at the Genomics Core at the University of Colorado Anschutz Medical Campus.

Raw metatranscriptome reads were quality trimmed, and adapters were removed using bbduk (87) with the following flags: k=23 mink=11 hdist=1 qtrim=rl trmiq=20 minlength=75. Reads were filtered with rqcfilter2 using the following flags: jni=t rna=t trimfragadapter=t qtrim=r trimq=0 maxns=1 maq=10 minlen=51 mlf=0.33 phix=t removeribo=t removehuman=t removedog=t removecat=t removemouse=t khist=t removemicrobes=t mtst=t sketch kapa=t clumpify=t tmpdir=null barcodefilter=f trimpolyg=5. Trimmed filtered reads were mapped using Bowtie2 (101) against the dereplicated MAG database (*n* = 1,864 MAGs) with the following settings: -D 10 -R 2 -N 1 -L 22 -I S,0,2.50. The resulting SAM file was converted to a BAM with samtools (107) and then filtered using the reformat.sh script from bbtools (87) with the following settings: idfilter=0.95 pairedonly=t primaryonly=t. Mapped reads were counted with htseq-count (108) with the flags: -a 0 -t CDS -I ID –stranded=reverse. Last, read counts were filtered to remove counts of <5 and were converted to geTMM values (109) in R. Metatranscriptomic reads are available on NCBI, with accession numbers reported in Table S1 tab MetaT_Accesion_Info.

### Further profiling of methylotrophy across Stordalen Mire MAGs

To better resolve the DRAM-suggested potential of the Stordalen methanogens for methylotrophy, the MAGs were searched via BLAST-P using a FASTA reference file (Fig. S2C; Table S2 tab FASTA_reference_for_genes_trees) of 53 well-characterized methylotrophic gene types (20 *mtxB* genes, 16 *mtxC* genes, 10 *mtxA* genes, and 7 *ram* genes). The BLAST-P output (Table S2 tab BLASTP_results) was limited to include only hits with a bitscore of >60 from MAGs found to encode homologs of *mtxB* genes. Genes passing this threshold were phylogenetically analyzed using ProtPipeliner to build RaxML trees (https://github.com/TheWrightonLab/Protpipeliner/blob/master/protpipeliner.py) relative to reference genes, including those used in the BLAST-P search, plus other homologous sequences derived from UniProt from physiologically characterized methylotrophic methanogens and acetogens (Table S2 tab FASTA_reference_for_genes_trees). Newick trees are available at https://doi.org/10.5281/zenodo.7864933. Gene trees were built for *mtxB* (127 genes), *mtxA* (168 genes), *mtxC* (190 genes), *ramX* (100 genes), and methylated sulfur genes (36 genes). Trees were visually inspected in iTOL (110), and tree placement was used to confirm or refine the specific identification of genes (Table S2 tab Gene_ID_per_trees). In some cases, genes were only ambiguously identified as methylotrophy-relevant but substrate-nonspecific *mtxA* or *mtxC*. RamX proteins are known to be promiscuous activating enzymes across corrinoid proteins (111), and so activase-encoding genes were identified as evidence for methylotrophy in conjunction with *mtxBCA* genes but not used to infer substrate specificity for MAGs.

Eighty-five of the 86 MAGs belonging to the *Methanobacteriales*, *Methanosarcinales,* and *Methanomassiliicoccales* were found to encode genes for methylotrophy and were used throughout the remaining analyses of methylotrophic methanogenesis. However, MAGs were conservatively defined as methylotrophic only if they were found to encode genes for at least two types of the three core members of a methyltransferase system directly involved in substrate demethylation (MtxB, MtxC, and MtxA), one of which had to be the substrate demethylating *mtxB*. In the case of methyl sulfide metabolism, MAGs were screened for having single genes encoding any of the tri-functional MtsDFH proteins or at least one of the *mtsAB* (see Fig. 1C; Fig. S2). To determine if methylotrophy was being expressed within the MAGs, identified methylotrophic genes were mined from paired metatranscriptomic data (Table S2 tab Methanogen_Methylotrophic_geTMM). Analogous rules were used to label MAGs as active methylotrophs only if they were found to be expressing the majority of an identified methyltransferase system, including an *mtxB* gene. To determine overall order-level methanogen field activity, average (*n* = 3) geTMM values for all methanogen-expressed genes were mined from the data (Table S2 tab Methanogen_geTMM_all_genes). Here, the *Methanomassiliicoccales* were identified as obligate likely hydrogen-dependent methylotrophs, while the *Methanobacteriales* and *Methanosarcinales* were classified as facultative methylotrophs.

Next, to query the bacterial community for methylotrophy genes, a similar BLAST-P approach was used to query the 12,868 bacterial MAGs just for *mtxB* genes—considered here the best single marker gene for methylotrophy—with a minimum bitscore of >200 (Table S5 tab BLAST_bitscore>200). A subset of the same (Table S2 tab FASTA_reference_for_genes_trees) FASTA reference file was used, limited to include only the *mtxB* genes. Bacterial MAGs were only screened for the highly substrate-specific *mtxB* as the best marker gene of methylotrophy to reduce potential nonspecific hits to other methylotrophy genes (e.g., other bacterial cobalamin-binding proteins). Identified genes were parsed from metatranscriptomes to determine active bacterial methylotrophy. Phylogenetic analysis using ProtPipeliner (Newick trees available at https://doi.org/10.5281/zenodo.7864933) to confirm the substrate-resolved identity of *mtxB* genes was performed only for those encoded and expressed by the *Acidobacteriota, Actinobacteriota*, and *Proteobacteria* represented in Fig. 5B. To distinguish between bacterial *mttB* genes encoding and lacking the unique pyrrolysine (Pyl) residue, sequences were analyzed using Geneious 2023.0.1 (www.geneious.com) to look for gene truncation due to the amber codon encoding Pyl. Those found to be truncated were identified as “Pyl-MttB” [known to be specific to tri/di/monomethylamine (54)], and those found to lack said truncation were identified as “Non-Pyl MttB/MtxB” [known to be specific for quaternary amines (30)] (Fig. 5B).

### MAG metagenome relative abundance determination

To determine metagenome abundances of the 97% dereplicated MAG set, we first mapped trimmed metagenome reads to the MAG set using Bowtie2 (v2.4.5) (101) using the following settings: -D 10 -R 2 -N 1 -L 22 -I S,0,2.50. The output SAM file was converted to a sorted BAM using samtools (v1.9) (112) and filtered using the reformat.sh script in the bbtools (87) package using idfilter=0.95 pairedonly=t primaryonly=t. MAG abundance was inferred from this using coverM (v0.6.0). The coverM (v0.6.0) genome (https://github.com/wwood/CoverM) was then run using the produced BAM files as input to calculate the coverage of the dereplicated MAGs within the field to permit the calculation of methanogen relative abundance. coverM was run with the following flags: coverm genome–-proper-pairs-only -x fna –min-read-percent-identity-pair 0.95 –min-read-alignment-percent-pair 0.75 -m trimmed-means –trim-min 0.1 –trim-max 0.9. MAG relative abundances were calculated using the output in R. A final table containing the relative abundance of all MAGs within the 97% dereplicated set, plus the abundances of methanogen MAGs normalized to both the total summed abundance of all archaea and all methanogens, is shown in Table S4.

### Phylogenomic analysis of the native methanogen community

Phylogenomic analysis of the 367 Stordalen Mire methanogen MAGs (Table S1 tab Methanogen_Genome_Info) was performed using the GTDB-Tk v2.1.1 r207 (104) run using the *de novo* workflow. The alignment was based on 53 concatenated archaeal marker genes, and a GTDB-derived genome from the phylum *Undinarchaeota* (GCA_002495465.1) was used as an outgroup to root the tree. The generated tree was read and visually modified in R using the ggtree package (113). The Newick tree is available at https://doi.org/10.5281/zenodo.7864933.

### Metabolite extraction and LC-MS/MS

Water-soluble metabolites were extracted from peat by adding 7 mL of autoclaved Milli-Q water to 1 g of wet peat in a sterile 15-mL centrifuge tube. Tubes were vortexed for 30 s two times, sonicated for 2 h at 22°C, and then centrifuged; the resulting 6-mL supernatant served as the water extract. Two milliliters of this was aliquoted for LC-MS/MS and stored at −80°C until use.

Water-soluble extracted metabolites were thawed at room temperature and centrifuged to remove any resultant particulates. Each sample was divided into two 1-mL aliquots in 2-mL glass tube vials for hydrophilic interaction liquid chromatography (HILIC) and reverse-phase (RP) liquid chromatography, respectively. Both vials were dried down completely on a Vacufuge plus (Eppendorf, USA). Samples for HILIC were resuspended in a 50:50 solution of acetonitrile and water. Samples for RP were resuspended in a 20:80 solution of HPLC grade methanol in water.

A Thermo Scientific Vanquish Duo ultra-high-performance liquid chromatography (UHPLC) system was used here for liquid chromatography. Extracts were separated with a Waters ACQUITY HSS T3 C18 column for RP separation and a Waters ACQUITY BEH amide column for HILIC separation.

Samples were injected in a 1-µL volume onto the column and eluted as follows: for RP, the gradient went from 99% mobile phase A (0.1% formic acid in H_2_O) to 95% mobile phase B (0.1% formic acid in methanol) over 16 min. For HILIC, the gradient went from 99% mobile phase A (0.1% formic acid, 10 mM ammonium acetate, 90% acetonitrile, and 10% H_2_O) to 95% mobile phase B (0.1% formic acid, 10 mM ammonium acetate, 50% acetonitrile, and 50% H_2_O). Both columns were run at 45°C with a flow rate of 300 µL/min.

A Thermo Scientific Orbitrap Exploris 480 was used for spectral data collection with a spray voltage of 3,500 V for positive mode (for RP) and 2,500 V for negative mode (for HILIC) using the H-ESI source. The ion transfer tube and vaporizer temperature were both 350°C. Compounds were fragmented using data-dependent MS/MS with higher energy collisional dissociation (HCD) collision energies of 20, 40, and 80.

The commercially available Compound Discoverer 3.2 software (Thermo Fisher Scientific) was used to analyze the data using the untargeted metabolomic workflow. Briefly, the spectra were first aligned, followed by a peak picking step. Putative elemental compositions of unknown compounds were predicted using the exact mass, isotopic pattern, fine isotopic pattern, and MS/MS data using the built-in HighChem Fragmentation Library of reference fragmentation mechanisms. Metabolite annotation was performed first by matching fragmentation scans, retention time, and ion mass to an in-house database built using 1,200 reference standards. Second, fragmentation scan (MS2) searches in mzCloud were performed, which is a curated database of MSn spectra containing more than 9 million spectra and 20,000 compounds.

Third, predicted compositions were obtained based on the mass error, matched isotopes, missing number of matched fragments, spectral similarity score (calculated by matching theoretical and measured isotope patterns), matched intensity percentage of the theoretical pattern, relevant portion of MS, and MS/MS scan. The mass tolerance used for estimating the predicted composition was 5 ppm. Finally, annotation was complemented by searching MS1 scans on different online databases with ChemSpider (using either the exact mass or the predicted formula). Based on the annotation results, metabolites were divided into four categories and were assigned as either Level 1, Level 2, or Level 3 following the Metabolomics Standards Initiative (114) : (i) full match to in-house databases, (ii) full match based on mzCloud, predicted composition, and ChemSpider, (iii) full match based on predicted composition and ChemSpider, and (iv) annotated by only one method (ChemSpider) with potential annotation being based on mass alone.

The structures of all chemical compounds identified via LC-MS/MS (Table S3 tabs 7–9) in Stordalen Mire were examined only for any rank 1 species, including a methylated nitrogen, oxygen, or sulfur atom. Methylated compounds that met this criterion were then compared to known methylotrophic substrates (Fig. S3A and B) to look for structural homology. Note that compounds of interest for this study were only identified in the RP and not the HILIC data, and thus, only the former is of focus in this manuscript. Peak areas for identified compounds were normalized to the sum of all compounds within each individual sample prior to further analysis in R. Boxplots made in R v4.1.1(115) (Fig. S3C) show the individual averaged (*n* = 3) LC-MS/MS peak area for each species over thaw and depth gradients; these averaged peak areas were categorically summed by “methyl-N”/methylated amines and “methyl-O”/methylated oxygen compounds, which is shown in Fig. 3B. LC-MS/MS data can be found at https://doi.org/10.5281/zenodo.7519815.

### ^1^H NMR

To identify field-present metabolites in Stordalen, including methanogenic and methylotrophic precursors, we performed ^1^H NMR on aliquots of the same peat extractions prepared for LC-MS/MS analyses. Samples (180  µL) were combined with 2,2-dimethyl-2-silapentane-5-sulfonate-d_6_ (DSS-d_6_) in D_2_O (20  µL, 5  mM) and thoroughly mixed prior to transfer to 3-mm NMR tubes. NMR spectra were acquired on a Varian 600 MHz VNMRS spectrometer equipped with a 5-mm triple-resonance (HCN) cold probe at a regulated temperature of 298K. The 90° ^1^H pulse was calibrated prior to the measurement of each sample. The one-dimensional (1D) ^1^H spectra were acquired using a nuclear Overhauser effect spectroscopy (NOESY) pulse sequence with a spectral width of 12 ppm and 512 transients. The NOESY mixing time was 100  ms, and the acquisition time was 4  s, followed by a relaxation delay of 1.5  s during which presaturation of the water signal was applied. Time-domain free induction decays (57,472 total points) were zero filled to 131,072 total points prior to Fourier transform. Chemical shifts were referenced to the ^1^H methyl signal in DSS-d_6_ at 0 ppm. The 1D ^1^H spectra were manually processed, assigned metabolite identification, and quantified using Chenomx NMR Suite 8.3. Metabolite identification was based on matching the chemical shift, J-coupling, and the intensity of experimental signals to compound signals in the Chenomx and custom in-house databases. Quantification was based on fitted metabolite signals relative to the internal standard (DSS-d_6_). Signal-to-noise ratios (S/N) were measured using MestReNova 14 with the limit of quantification equal to an S/N of 10 and the limit of detection equal to an S/N of 3. Known methanogenic substrates were manually identified from the list of quantitated metabolites, and average (*n* = 3) concentrations were plotted using R v4.1.1(115), as shown in Fig. 3. Summarized data are available in Table S3, and raw data can be found at https://doi.org/10.5281/zenodo.7519683.

### Data visualization and statistics

Data were analyzed and visualized in R v4.1.1 (115, 116) using ggplot2 (117) unless otherwise specified. The superheat package was used to generate the heatmap in Fig. 1D. All reported statistical analyses, including ANOVA, Tukey’s HSD, and Pearson’s correlation, were performed in R using the ggpubr package (118).

## Data Availability

The metagenomes, metatranscriptomes, and metagenome-assembled genomes used in this paper are available at NCBI under BioProjectID PRJNA386568 (43). Table S1 provides individual BioSample numbers. Raw and processed data for all analyses are available in the following Zenodo archives: 97% dereplicated MAG annotations (10.5281/zenodo.7587534), metatranscriptome mapping (10.5281/zenodo.7591900), LC-MS/MS (10.5281/zenodo.7519815), NMR (10.5281/zenodo.7519683), July 2016 methane flux data (10.5281/zenodo.7897922), and Newick trees for phylogenomic and phylogenetic analyses plus DRAM v1.3.2 annotations for all methanogens and methylotrophic bacteria (10.5281/zenodo.7864933). MAGs have been submitted to NCBI under accession number PRJNA386568. Photographs of the Mire used in Fig. S1 [file names: AJ_palsa_05.jpg, Eriophorum_eample_06.jpg, Sphagnum_04_(drier).jpg] were all taken by Nicole Raab in July 2016 and retrieved/available from the EMERGE database (emerge-db.asc.ohio-state.edu). Last, the full field metadata sheet for Stordalen Mire published by EMERGE is available at 10.5281/zenodo.7720573.

## SUPPLEMENTAL MATERIAL

Figure S1 msystems.00698-23-s0001.tif Sampling design with pictures and map of site.

Figure S2 msystems.00698-23-s0002.tif Details of methanogen MAG physiological curation.

Figure S3 msystems.00698-23-s0003.tif Genes and substrates known involved in methylotrophy, plus potential substrates present in Stordalen Mire.

Figure S4 msystems.00698-23-s0004.tif Curation of the proportion of methanogen order-level activity due to methylotrophic MAGs.

Figure S5 msystems.00698-23-s0005.tif Comparison of methylotrophic bacteria and methanogen activity.

Table S1 msystems.00698-23-s0006.xlsx MAG data plus sequence accession data.

Table S2 msystems.00698-23-s0007.xlsx Data on genomic and physiological curation of methanogens.

Table S3 msystems.00698-23-s0008.xlsx Field chemistry data from Stordalen Mire.

Table S4 msystems.00698-23-s0009.xlsx Relative abundance table of methanogen MAGs within metaG and metaT data.

Table S5 msystems.00698-23-s0010.xlsx Genomic and physiological curation of methylotrophic bacterial MAGs.

ASM does not own the copyrights to Supplemental Material that may be linked to, or accessed through, an article. The authors have granted ASM a non-exclusive, world-wide license to publish the Supplemental Material files. Please contact the corresponding author directly for reuse.
