# Supplementary figures and images for: Methylotrophy in the Mire: direct and indirect routes for methane production in thawing permafrost
Source: mSystems. 2023 Dec 8;9(1):e00698-23. doi: 10.1128/msystems.00698-23 (PMC10805028; doi:10.1128/msystems.00698-23)

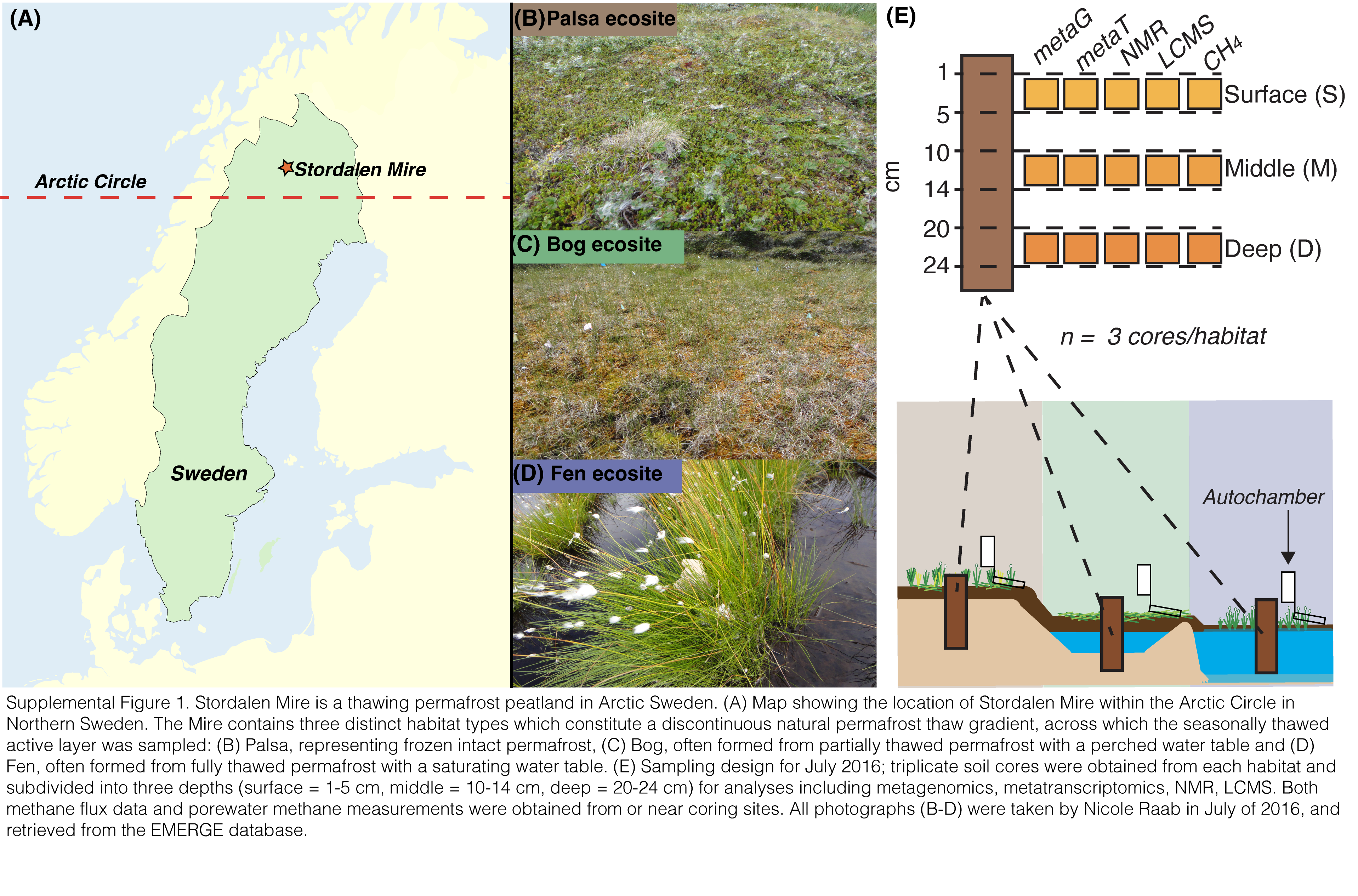

Supplement: Figure S1 — Sampling design with pictures and map of site. [file msystems.00698-23-s0001.tif]

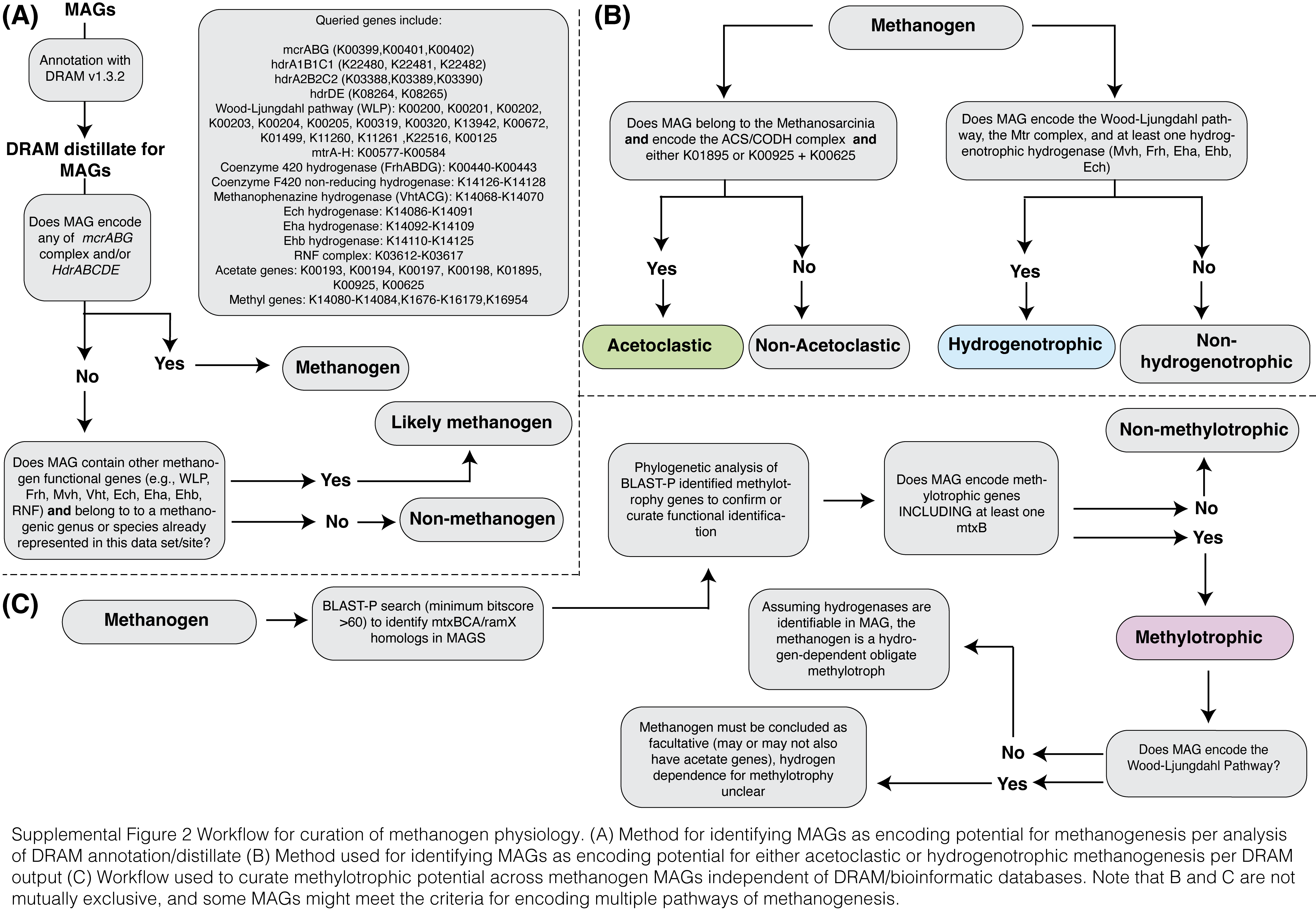

Supplement: Figure S2 — Details of methanogen MAG physiological curation. [file msystems.00698-23-s0002.tif]

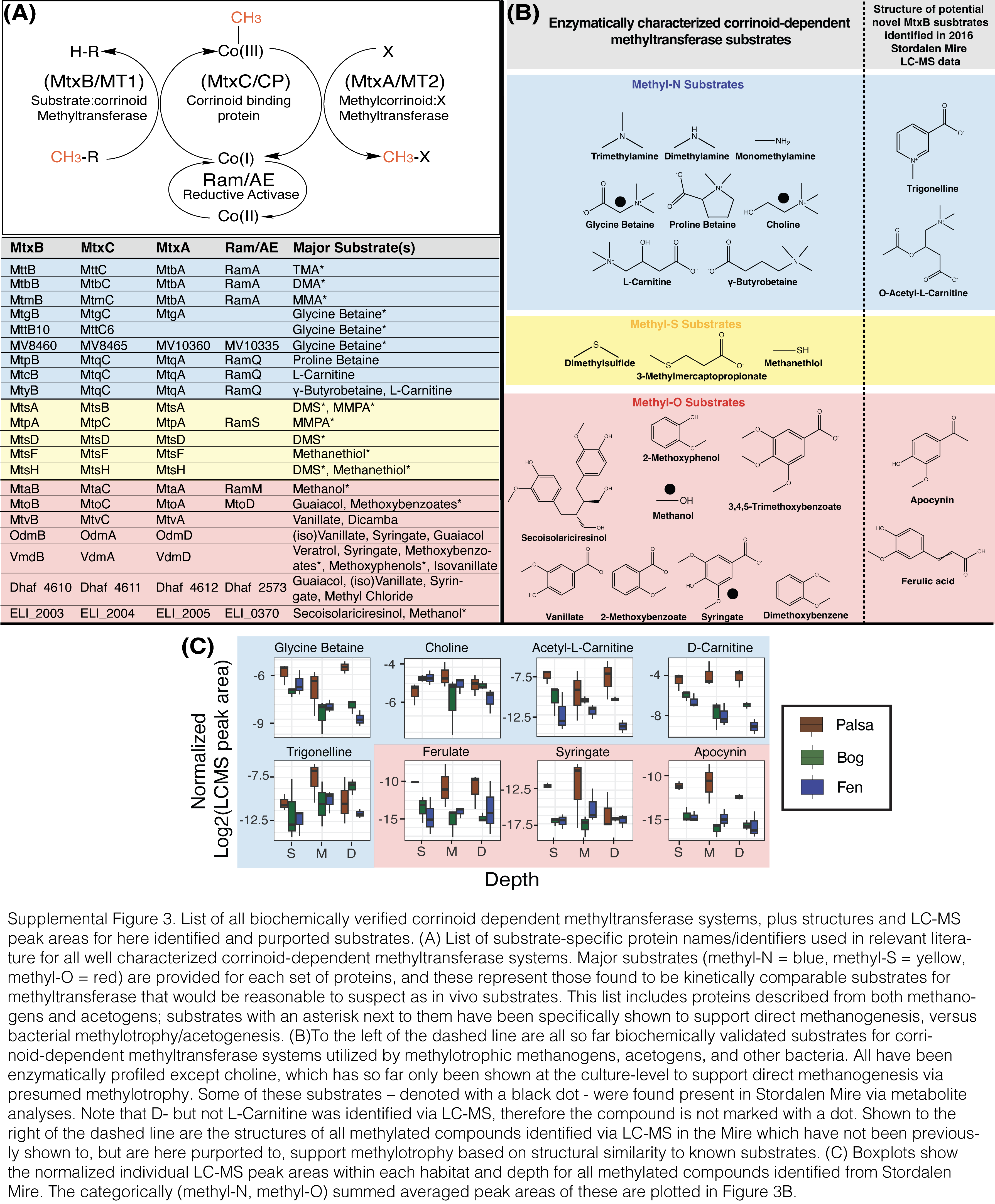

Supplement: Figure S3 — Genes and substrates known involved in methylotrophy, plus potential substrates present in Stordalen Mire. [file msystems.00698-23-s0003.tif]

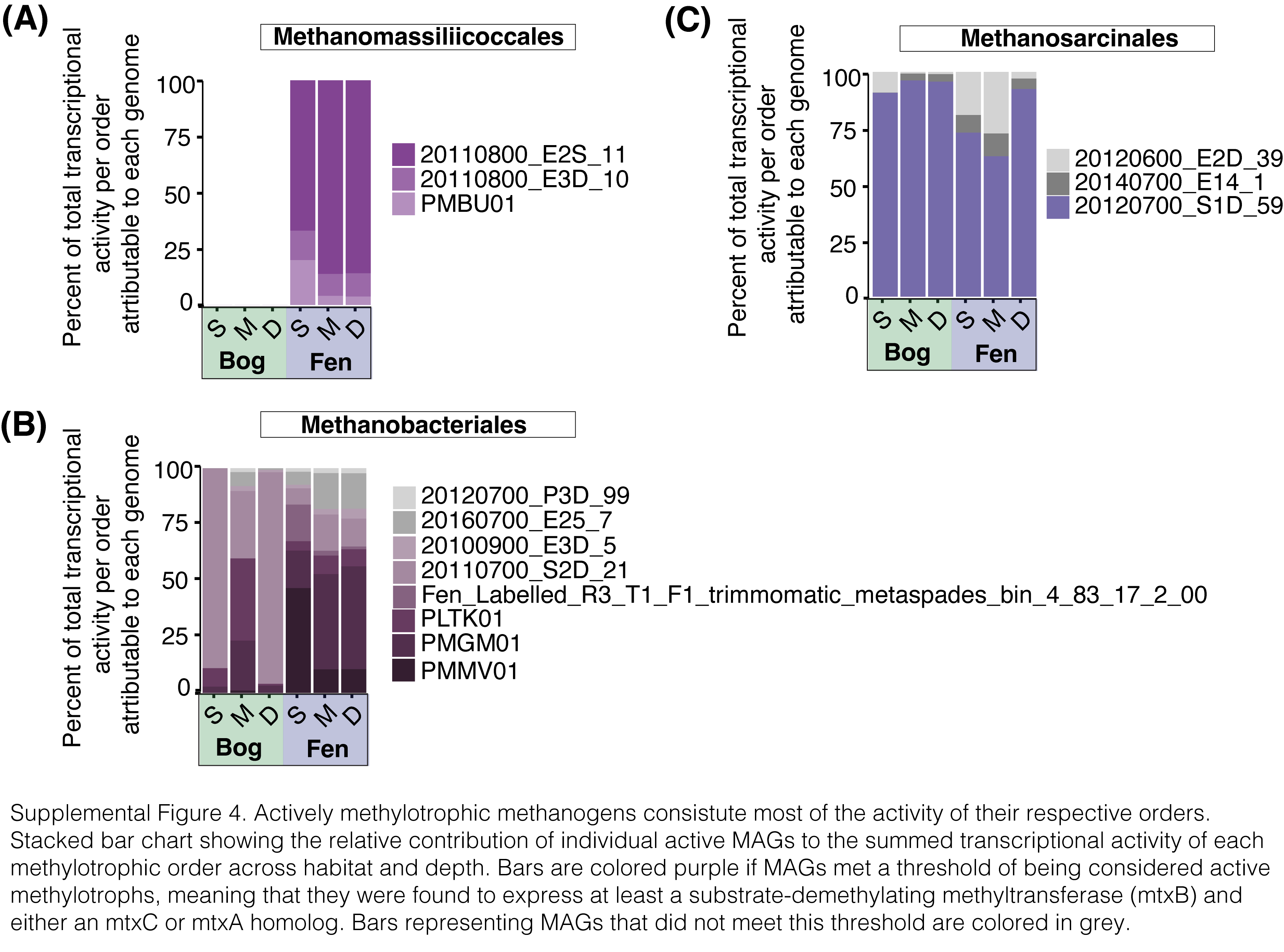

Supplement: Figure S4 — Curation of the proportion of methanogen order-level activity due to methylotrophic MAGs. [file msystems.00698-23-s0004.tif]

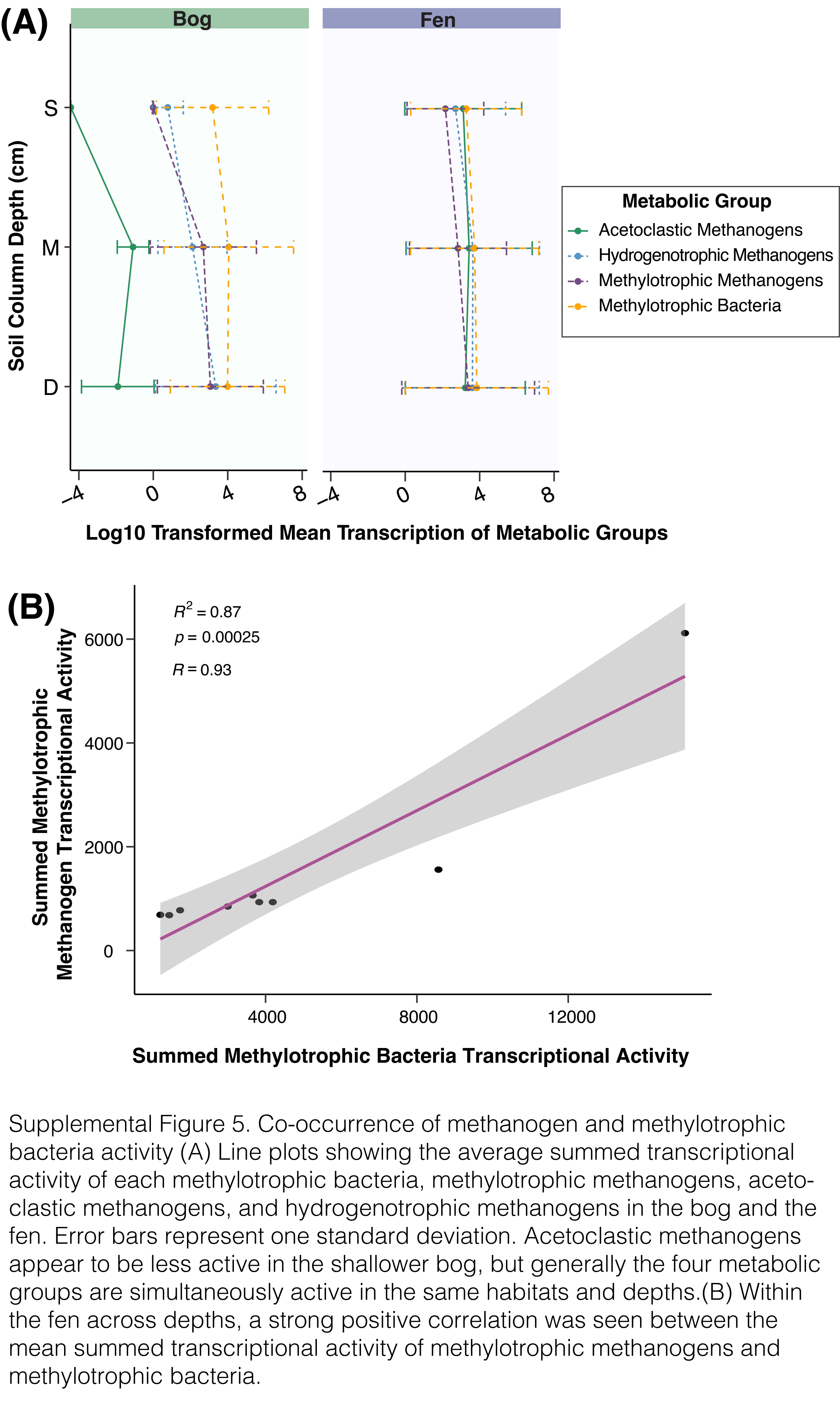

Supplement: Figure S5 — Comparison of methylotrophic bacteria and methanogen activity. [file msystems.00698-23-s0005.tif]
